# Epigenetic treatment of behavioral and physiological deficits in a tauopathy mouse model

**DOI:** 10.1111/acel.13456

**Published:** 2021-09-21

**Authors:** Wei Wang, Qing Cao, Tao Tan, Fengwei Yang, Jamal B. Williams, Zhen Yan

**Affiliations:** ^1^ Department of Physiology and Biophysics Jacobs School of Medicine and Biomedical Sciences State University of New York at Buffalo Buffalo New York USA

**Keywords:** Alzheimer’s disease, EHMT, excitability, histone methyltransferase, memory, prefrontal cortex, synaptic function, Tau

## Abstract

Epigenetic abnormality is implicated in neurodegenerative diseases associated with cognitive deficits, such as Alzheimer's disease (AD). A common feature of AD is the accumulation of neurofibrillary tangles composed of hyperphosphorylated tau. Transgenic mice expressing mutant P301S human tau protein develop AD‐like progressive tau pathology and cognitive impairment. Here, we show that the euchromatic histone‐lysine N‐methyltransferase 2 (EHMT2) is significantly elevated in the prefrontal cortex (PFC) of P301S Tau mice (5–7 months old), leading to the increased repressive histone mark, H3K9me2, which is reversed by treatment with the selective EHMT inhibitor UNC0642. Behavioral assays show that UNC0642 treatment induces the robust rescue of spatial and recognition memory deficits in P301S Tau mice. Concomitantly, the diminished PFC neuronal excitability and glutamatergic synaptic transmission in P301S Tau mice are also normalized by UNC0642 treatment. In addition, EHMT inhibition dramatically attenuates the hyperphosphorylated tau level in PFC of P301S Tau mice. Transcriptomic analysis reveals that UNC0642 treatment of P301S Tau mice has normalized a number of dysregulated genes in PFC, which are enriched in cytoskeleton and extracellular matrix organization, ion channels and transporters, receptor signaling, and stress responses. Together, these data suggest that targeting histone methylation enzymes to adjust gene expression could be used to treat cognitive and synaptic deficits in neurodegenerative diseases linked to tauopathies.

## Graphical abstract

In prefrontal cortical neurons of Alzheimer's disease, EHMT and H3K9me2 levels are elevated, hyperphosphorylated tau causes microtubule (MT) disintegration, leading to reduced synaptic strength and cognitive impairment (top panel). After treatment with the EHMT inhibitor UNC0642, H3K9me2 level is normalized, tau phosphorylation is diminished, and MT is stabilized, leading to enhanced synaptic strength and cognitive improvement (bottom panel).

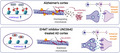

## 1 INTRODUCTION

Intracellular accumulation of neurofibrillary tau tangles is the common hallmark of Alzheimer's disease (AD; Dubois et al., 2014). Tau, a microtubule (MT)­associated protein, is critically involved in MT assemble and stabilization. Normal phosphorylation of tau controls the dynamics of MT, establishing neuronal polarity, axonal outgrowth, and axonal transport (Caceres & Kosik, 1990; Harada et al., 1994; Wang & Mandelkow, 2016). Abnormally hyperphosphorylated tau assembles tau into tangles of filaments and breaks down MTs, which disrupts synaptic functions and leads to synapse loss correlated with cognitive impairment prior to overt neurodegeneration (Alonso et al., 1996; Hoover et al., 2010; Mielke et al., 2017). However, there are currently limited treatment options for tau pathology‐linked disorders including AD.

Emerging evidence shows that epigenetic factors that integrate environmental factors (e.g., aging) into chromatin remodeling can alter neuronal functions, which contributes to the cognitive decline linked to neurodegenerative diseases (Nativio et al., 2018; Sen et al., 2016). Controlling gene expression through epigenetic regulation, including chromatin remodeling and histone modification, provides a potential avenue to restore synaptic and cognitive plasticity in AD (Li et al., 2019; Yuan et al., 2020).

A key epigenetic regulator of neuronal function, the euchromatichistone‐lysine N‐methyltransferase (EHMT), which represses genes through histone 3 lysine 9 (H3K9) methylation (Tachibana et al., 2005), targets most of genes involved in learning and memory in *Drosophila* (Kramer et al., 2011). Human databases have shown that the expression of EHMT1 in the frontal cortex increases with age and positively correlates with AD progression (Lu et al., 2004; Sharma et al., 2017; Yuan et al., 2020). Ablation of the negative regulator, H3K9 methylation, prevents Aβ‐induced plasticity deficits in hippocampal neurons (Sharma et al., 2017), improves cognitive behavior in an Aβ‐linked familial AD model (Zheng et al., 2019) mitochondrial function in aging mice (Yuan et al., 2020).

The therapeutic potential of targeting EHMT to treat tau‐mediated neurodegenerative disorders remains unknown. To address this, we used a transgenic mouse model expressing human tau bearing P301S mutation, which develops filamentous tau lesions at 6 months of age (Yoshiyama et al., 2007; Zhang et al., 2014). Prefrontal cortex (PFC), a key target region of AD that is critical for high‐level cognitive function (Maillet & Rajah, 2013; Spellman et al., 2015; Tan et al., 2021; Yan & Rein, 2021), was focused. We discovered that euchromatic histone‐lysine *N*‐methyltransferase 2 (EHMT2) was significantly elevated in PFC of P301S Tau mice, and inhibition of EHMT led to the amelioration of behavioral and synaptic deficits, as well as the normalization of large‐scale gene expression. It supports the potential of EHMT as a target for the treatment of tauopathies, including AD.

## 2 RESULTS

### 2.1 P301S Tau transgenic mice exhibit the increased level of EHMT2 and H3K9me2, and EHMT inhibition rescues their memory deficits

**FIGURE 1:**
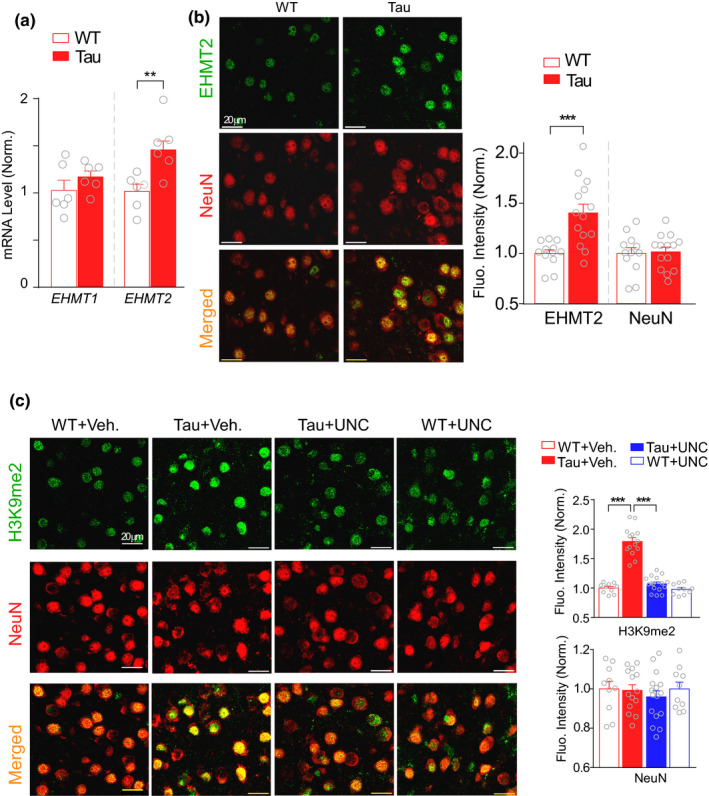
EHMT2 and H3K9me2 levels are significantly elevated in PFC neurons of P301S Tau mice. (a) Bar graphs showing *EHMT1* and *EHMT2* mRNA levels in PFC from WT and P301S Tau mice (**p < 0.01, *t* test). (b) Representative confocal images and quantification of the intensity of EHMT2 and NeuN immunofluorescent signals in PFC from WT vs. P301S Tau mice (***p < 0.001, *t* test). Merged images show the colocalization of EHMT2 and NeuN. (c) Representative confocal images and quantification of the intensity of H3K9me2 and NeuN signals in PFC from WT versus P301S Tau mice treated with EHMT inhibitor UNC0642 (1 mg/kg, i.p., 3x) or vehicle control (***p < 0.001, two‐way ANOVA). Merged images show the colocalization of H3K9me2 and NeuN

Given the positive correlation of increased EHMT1 in the human frontal cortex with AD progression (Lu et al., 2004; Sharma et al., 2017; Yuan et al., 2020; Zheng et al., 2019), we examined the level of EHMT1 (GLP) and EHMT2 (G9a) in the PFC of an AD‐associated mouse model, P301S Tau mice (Mathys et al., 2019; Yoshiyama et al., 2007; Zhang et al., 2014). Quantitative PCR data showed that the mRNA level of EHMT2 in the PFC slices from P301S Tau mice (~6 months old) was significantly higher than that from WT mice (Figure 1a, *n* = 6 pairs, *t*_(10)_ = 3.6, *p* = 0.0045, *t* test), while the mRNA level of EHMT1 was largely unchanged. Immunostaining of brain slices demonstrated that EHMT2 level in PFC neurons (NeuN+) from P301S Tau mice (~6 months old) was significantly increased (Figure 1b, WT: 1.0 ± 0.04, Tau: 1.40 ± 0.09, *n* = 12–14 slices from 2 to 3 mice each group, *t*_(24)_ = 4.0, *p* < 0.001, *t* test). EHMT1/2 specifically catalyzes H3K9 dimethylation (H3K9me2). We found that the level of H3K9me2 was significantly elevated in PFC neurons (NeuN+) of P301S Tau mice (Figure 1c). Treatment with UNC0642 (1 mg/kg, i.p., once daily for 3 days), a brain‐penetrant, highly selective and potent EHMT1/2 inhibitor (Kim et al., 2017; Liu et al., 2013), significantly reduced the elevated H3K9me2 level in P301S Tau mice (Figure 1c, *n* = 10–16 slices, 2–4 mice/group, *F*_1,46(interaction)_ = 58.3, *p* < 0.001, *F*_1,46(genotype)_ = 92.7, *p* < 0.001, *F*_1,46(treatment)_ = 65.5, *p* < 0.001; *p* < 0.001, two‐way ANOVA). No significant changes were found in the number of NeuN + neurons (*p* > 0.05, *t* test), suggesting the lack of obvious neuronal loss at this age of Tau mice.

**FIGURE 2:**
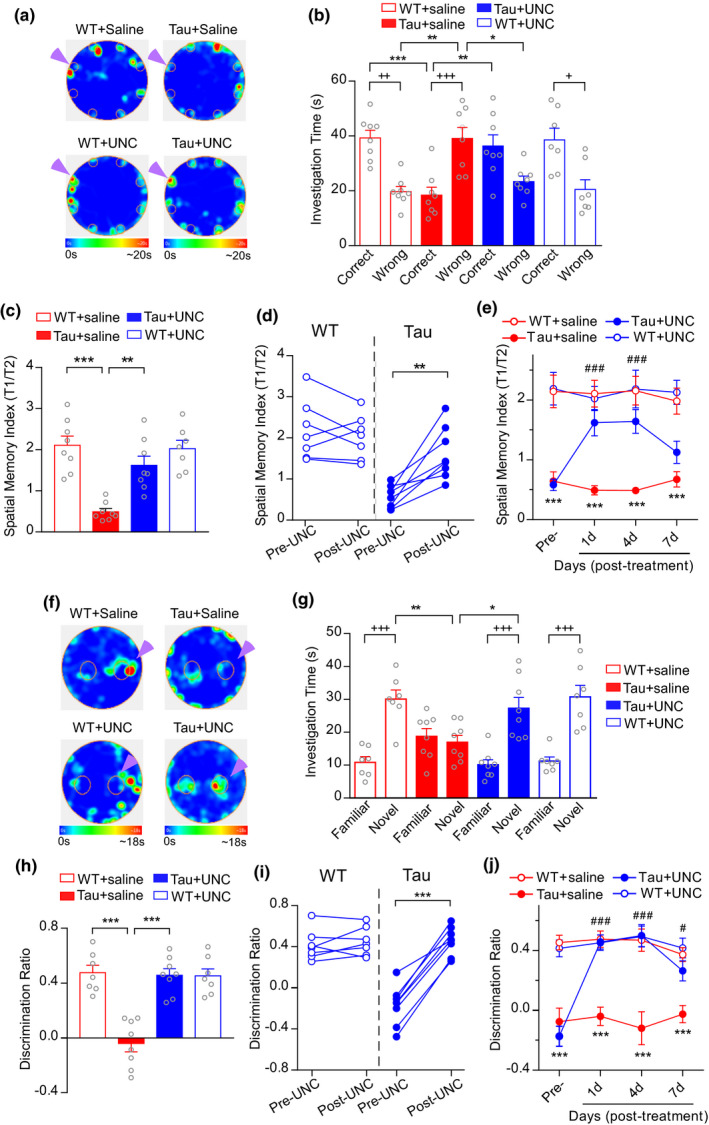
Administration of EHMT inhibitor UNC0642 improves cognitive function in P301S Tau mice. (a) Heat maps depicting the topographical time distribution in BM tests of WT versus P301S Tau mice treated with UNC0642 (1 mg/kg, i.p., 3x) or saline. Locations of the correct holes are labeled with arrowheads. (b, c) Bar graphs showing the investigation time (T1: on the correct hole; T2: on the seven incorrect holes) (b) and the spatial memory index (T1/T2) (c) during BM tests of all groups (b: ^++^ *p* < 0.01, ^+++^ *p* < 0.001, T1 vs. T2; *p < 0.05, **p < 0.01, ***p < 0.001, two‐way ANOVA; c: **p < 0.01, ***p < 0.001, two‐way ANOVA). (d) Scatter plots showing the BM spatial memory index of individual mice pre‐ and post‐treatment of UNC0642 (**p < 0.01, paired *t* test). (e) Plots of BM spatial memory index in WT versus Tau mice treated with UNC0642 or saline at different days (***p < 0.001, WT + saline vs. Tau + saline; ^###^ *p* < 0.001, Tau + saline vs. Tau + UNC, two‐way rmANOVA). (f) Heat maps depicting the topographical time distribution in NOR tests of WT versus Tau mice treated with UNC0642 or saline. Locations of novel objects are labeled with arrowheads. (g, h) Bar graphs showing the exploration time (*T*_Fam_: on the familiar object; *T*_Nov_: on the novel object) (g) and the discrimination ratio (h) during NOR tests of all groups (g: ^+++^ *p* < 0.001, *T*_Fam_ vs. *T*_Nov_; *p < 0.05, **p < 0.01, two‐way ANOVA; h: ***p < 0.001, two‐way ANOVA). (i) Scatter plots showing the NOR discrimination ratio in individual mice pre‐ and post‐treatment of UNC0642 (***p < 0.001, paired *t* test). (j) Plots of NOR discrimination ratio in WT and Tau mice treated with UNC0642 or saline at different days (***p < 0.001, WT + saline vs. Tau + saline; ^#^ *p* < 0.05, ^###^ *p* < 0.001, Tau + saline vs. Tau + UNC, two‐way rmANOVA). Data in figures (a–d) and (f–i) were collected at day 1 after cessation of UNC0642 treatment

Given the elevation of EHMT2, we next tested whether EHMT inhibition could alleviate cognitive deficits in P301S Tau mice (5–7 months old). We first performed Barnes maze (BM), an assay to test the animal's retention and retrieval of spatial memory by recalling the location of one correct hole (where an escape box was attached in the training phases) from seven other incorrect holes on a round platform based on visual cues (Zheng et al., 2019). As shown in Figure 2a,b, compared with WT mice, P301 Tau mice spent significantly less time exploring the correct hole (T1) and more time exploring the seven incorrect holes (T2), while UNC0642‐treated Tau mice had significantly increased time on the correct hole and decreased time on the incorrect holes (*n* = 7–8 mice/group, *F*_3,54(interaction)_ = 17.05, *p* < 0.0001, two‐way ANOVA). Accordingly, the spatial memory index (T1/T2) was markedly lower in P301S Tau mice than WT animals, which was significantly elevated by UNC0642 treatment (Figure 2c, *F*_1,27(interaction)_ = 10.03, *p* = 0.0038, two‐way ANOVA). Consistent improvement on spatial memory was observed in almost every examined P301S Tau mouse treated with UNC0642 (Figure 2d, *t*_(7)_ = 4.71, *p* = 0.0022, paired *t* test). The therapeutic effect of a brief UNC0642 administration on spatial memory deficits in P301S Tau mice sustained at least 4 days after the cessation of treatment (Figure 2e, *n* = 7–8 mice/group, *F*_9,81(interaction)_ = 3.34, *p* = 0.0016, two‐way rmANOVA).

We next conducted the novel object recognition (NOR) task, an assay to test recognition memory by measuring the exploration time spent on a novel object versus a familiar one. As shown in Figure 2f‐h, WT mice exhibited significantly longer investigation time on the novel objects than the familiar objects in NOR test, whereas P301S Tau transgenic mice showed no discrimination between the novel and familiar objects, and this recognition memory impairment in P301S Tau mice was markedly rescued by UNC0642 treatment (*n* = 7–8 mice/group, H: *F*_3,52(interaction)_ = 9.17, *p* < 0.001, two‐way ANOVA; I: *F*_1,26(interaction)_ = 22.66, *p* < 0.001, two‐way ANOVA). The elevated discrimination ratio induced by UNC0642 was consistently detected in individual P301S Tau mice (Figure 2i, *t*_(7)_ = 12.76, *p* < 0.0001, paired *t* test). Moreover, the rescue effect persisted for ~4 days and weakened at 7 days after the cessation of treatment (Figure 2j, *n* = 7–8 mice/group, *F*_9,78(interaction)_ = 5.99, *p* < 0.001, two‐way rmANOVA). UNC0642‐treated WT mice did not change the examined behaviors (Figure 2a‐j). Together, these results suggest that inhibition of the elevated EHMT2 can improve spatial and recognition memory in the tauopathy model of AD.

### 2.2 EHMT inhibition normalizes the excitability of PFC pyramidal neurons in P301S Tau mice

Next, we explored the physiological basis for the behavioral effects of EHMT inhibition. PFC is a brain region strongly linked to spatial memory retrieval and learning, as well as object recognition behaviors (Yan & Rein, 2021). The activity of PFC pyramidal neurons and recurrent excitation underlie PFC‐mediated cognitive function (Goldman‐Rakic, 1995), and loss of cortical network function contributes to cognitive decline in tauopathies (Menkes‐Caspi et al., 2015). Thus, we carried out current‐clamp recordings to measure the excitability of layer five PFC principal neurons.

**FIGURE 3:**
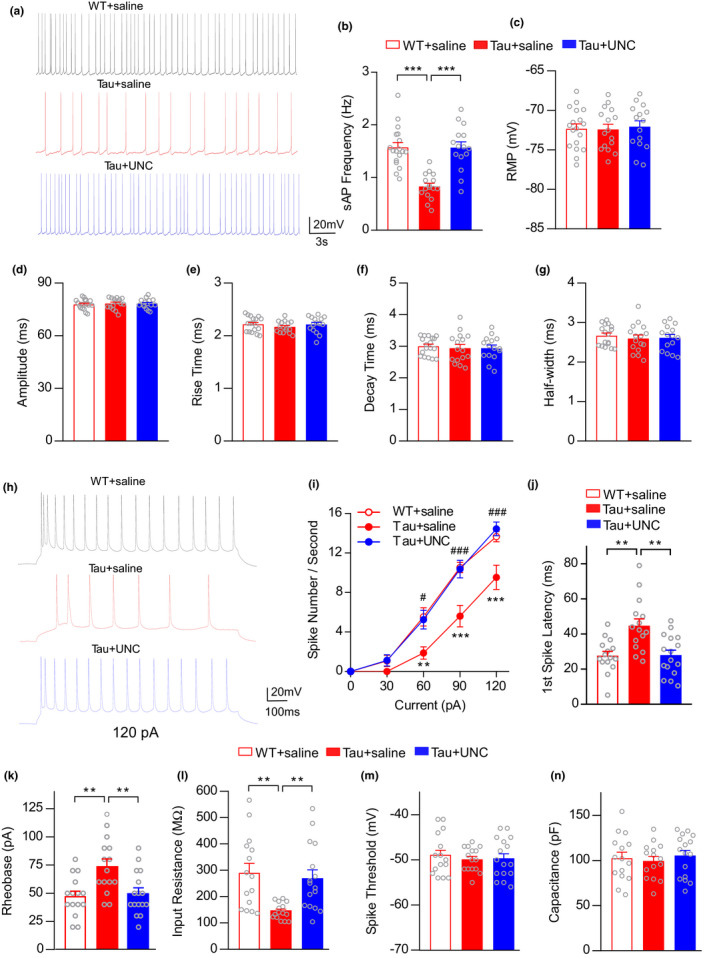
EHMT inhibitor UNC0642 rescues the diminished excitability of PFC pyramidal neurons in P301S Tau mice. (a) Synaptic‐driven spontaneous action potential (sAP) traces in PFC layer 5 (L5) pyramidal neurons from WT or P301S Tau mice (5–7 months old) treated with UNC0642 (1 mg/kg, i.p., 3x) or saline. (b‐g) Bar graphs showing firing frequency (b, *p* < 0.0001, one‐way ANOVA), RMP (c), sAP amplitude (d), rise time (e), decay time (f) and half‐width (g) in PFC L5 principal neurons. (h) Examples of evoked action potential (eAP) in responses to an injected current (120 pA) from PFC L5 pyramidal neurons of saline‐ or UNC0642‐injected WT or P301S Tau mice. (i) Plot of numbers of action potentials evoked by different current steps (**p < 0.01, ***p < 0.001, WT + saline vs. Tau + saline; ^#^ *p* < 0.05, ^###^ *p* < 0.001, Tau + saline vs. Tau + UNC, two‐way rmANOVA). (j‐n) Bar graphs showing the first spike latency of eAP (j), rheobase current (k), resistance (l), spike threshold (m), and capacitance (n) (**p < 0.01, one‐way ANOVA)

We first measured the synaptic‐driven spontaneous action potentials (sAP) using a low Mg^2+^ condition to enhance the activity of brain slices (Tan et al., 2021). As shown in Figure 3a,b, compared with age‐matched WT mice, sAP frequency of PFC pyramidal neurons was substantially lower in P301S mice (5–7 months old), which was significantly increased to the normal level by UNC0642 treatment (1 mg/kg, i.p., 3x) (*n* = 15–18 cells, 3–5 mice/group, *F*_2,46_ = 22.98, *p* < 0.0001, one‐way ANOVA). Resting membrane potential (RMP) and other sAP properties, including amplitude, rise time, decay time, and half‐width, did not change significantly (Figure 3c–g).

We further examined neuronal excitability by measuring action potentials elicited by injecting a series of depolarizing currents (eAP). As shown in Figure 3h,i, the frequency of evoked spikes was significantly lower in P301S Tau mice than WT mice and was reversed by UNC0642 treatment (*F*_2,43 (group)_ = 10.71, *p* = 0.0002, two‐way rmANOVA). Two other measures of excitability, the first spike latency, which plays an essential role in integrating synaptic events and influences the firing probability (Molineux et al., 2005), and rheobase, the minimum current required to elicit APs, were also compared. As shown in Figure 3j,k, both parameters were significantly increased in P301S Tau mice, and normalized by UNC0642 treatment (*n* = 15–16 cells, 3–4 mice/group, latency: *F*_2,43_ = 9.57, *p* = 0.0004; rheobase: *F*_2,43_ = 7.68, *p* = 0.0014, one‐way ANOVA). In addition, UNC0642 restored the significantly reduced input resistance in P301S Tau mice (Figure 3l, *F*_2,43_ = 7.78, *p* = 0.0013, one‐way ANOVA). Spike threshold and membrane capacitance were not significantly changed (Figure 3m,n). Overall, these data indicate that EHMT2 inhibition can rescue the diminished excitability of PFC pyramidal neurons in P301S Tau mice.

### 2.3 EHMT inhibition restores synaptic function and attenuates hyperphosphorylated tau in PFC of P301S Tau mice

**FIGURE 4:**
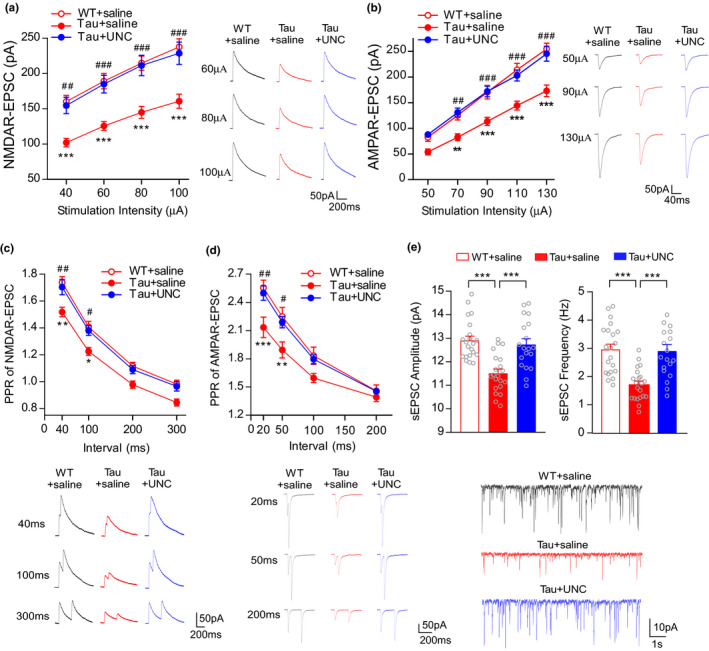
EHMT inhibitor UNC0642 restores NMDAR‐ and AMPAR‐mediated synaptic function in PFC of P301S Tau mice. (a, b) Input–output curves of NMDAR‐EPSC (a) and AMPAR‐EPSC (b) in layer 5 PFC pyramidal neurons from WT mice vs. P301S Tau mice (5–7 months old) treated with UNC0642 (1 mg/kg, i.p., 3x) or saline. Inset: representative NMDAR‐ and AMPAR‐EPSC traces at different stimuli (**p < 0.01, ***p < 0.001, WT + saline vs. Tau + saline; ^##^ *p* < 0.01, ^###^ *p* < 0.001, Tau + saline vs. Tau + UNC, two‐way rmANOVA). (c, d) Plot of PPRs of NMDAR‐EPSC (c) and AMPAR‐EPSC (d) evoked by double pulses across multiple intervals (20–300 ms) from PFC L5 pyramidal neurons of UNC0642‐ or saline‐treated WT versus P301S Tau mice. Inset: representative traces at different intervals (*p < 0.05, **p < 0.01, ***p < 0.001, WT + saline vs. Tau + saline; ^#^ *p* < 0.05, ^##^ *p* < 0.01, Tau + saline vs. Tau + UNC, two‐way rmANOVA). (e) Bar graphs showing the amplitude and frequency of sEPSC in WT vs. P301S Tau mice treated with UNC0642 or saline (***p < 0.001, one‐way ANOVA). Inset: sEPSC traces. Recordings were performed 1–4 days post‐injection

The excitability of PFC pyramidal neurons is mainly controlled by glutamatergic transmission. Synaptic dysfunction is a primary feature of AD and the basis of cognitive impairment (Styr & Slutsky, 2018). To assess the effect of UNC0642 on synaptic function, we next measured NMDAR‐ and AMPAR‐EPSC in layer 5 PFC pyramidal neurons from WT and P301S Tau mice (6–8 months old). As shown in Figure 4a,b, compared with WT mice, the amplitudes of NMDA‐EPSC and AMPAR‐EPSC evoked by various stimulation intensities were substantially smaller in P301S Tau mice, which were brought to the normal levels by UNC0642 treatment (NMDA: *n* = 17–22 cells, four mice/group, *F*_2,57 (group)_ = 13.42, *p* < 0.0001; AMPA: *n* = 17–20 cells, four mice/group, *F*_2,53(group)_ = 16.37, *p* < 0.0001, two‐way rmANOVA). The paired‐pulse ratios (PPRs) of NMDAR‐ and AMPAR‐EPSC across multiple inter‐stimulus intervals, an index of presynaptic release, were also significantly lower in P301S Tau mice, and were restored in UNC0642‐treated P301S Tau mice (Figure 4c,d, NMDA: *n* = 12–16 cells, 3–4 mice/group, *F*_2,38 (group)_ = 8.074, *p* = 0.0012; AMPA: *n* = 16–17 cells, four mice/group, *F*_2,47 (group)_ = 7.112, *p* = 0.002, two‐way rmANOVA). Furthermore, the amplitude and frequency of spontaneous EPSC (sEPSC) were significantly decreased in P301S Tau mice, which was markedly elevated by administration of UNC0642 (Figure 4e, *n* = 19–21 cells, four mice/group, Amplitude: *F*_2,58_ = 15.02, *p* < 0.0001; Frequency: *F*_2,58_ = 15.1, *p* < 0.0001, one‐way ANOVA). Together, the results suggest that the diminished glutamatergic transmission in P301S Tau mice may result from decreased presynaptic transmitter release and reduced postsynaptic receptors, and these synaptic deficits are rescued by inhibition of EHMT.

**FIGURE 5:**
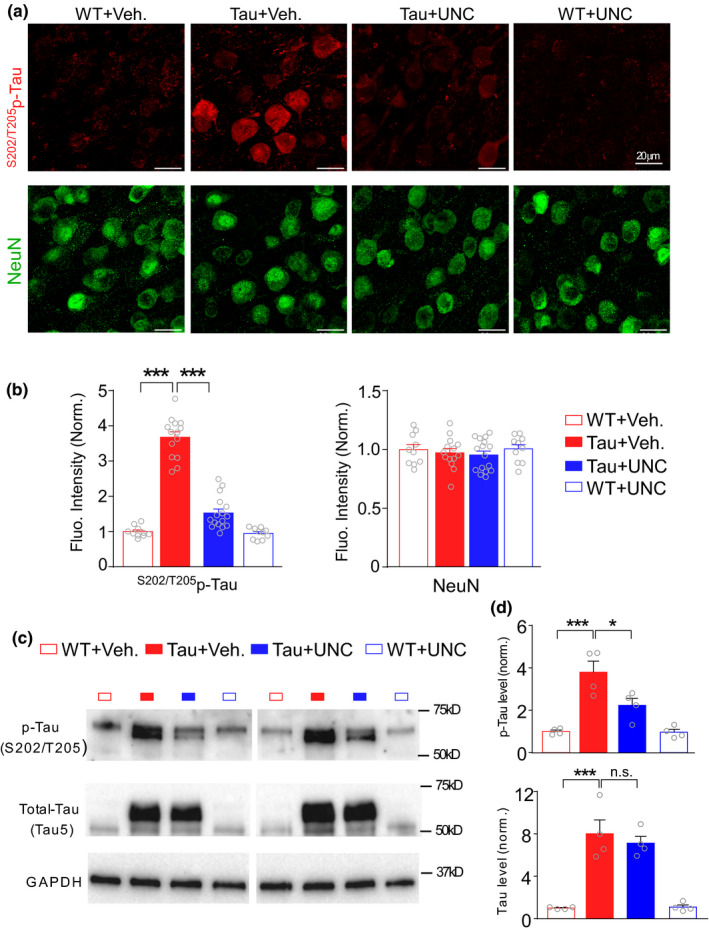
EHMT inhibitor UNC0642 reduces hyperphosphorylated tau in PFC of P301S Tau mice. (a, b) Representative confocal images (a) and quantification (b) of the fluoresce intensity of ^S202/T205^p‐tau (red) and NeuN (green) in PFC from WT vs. P301S Tau mice treated with UNC0642 (1 mg/kg, i.p., 3x) or vehicle control (***p < 0.001, two‐way ANOVA). (c, d) Representative Western blots (c) and quantification (d) of ^S202/T205^p‐tau and total tau signals in PFC from WT versus P301S Tau mice treated with UNC0642 or vehicle control (*p < 0.05, ***p < 0.001, two‐way ANOVA)

**FIGURE 6:**
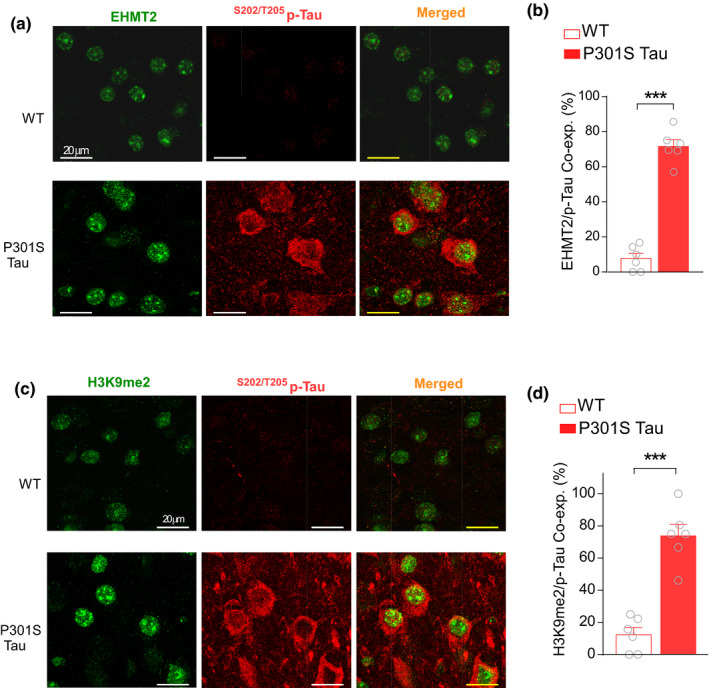
Hyperphosphorylated tau is co‐expressed with EHMT2 and H3K9me2 in PFC neurons of P301S Tau mice. (a, c) Confocal images of co‐staining of EHMT2 and ^S202/T205^p‐tau (a) or H3K9me2 and ^S202/T205^p‐tau (c) in PFC neurons from WT vs. P301S Tau mice. (b, d) Bar graphs showing the percentage of PFC neurons co‐expressing EHMT2 and p‐tau (b) or H3K9me2 and p‐tau (d) in WT versus P301S Tau mice

A pathological hallmark of tauopathy is the presence of hyperphosphorylated tau (p‐Tau). Next, we investigated whether EHMT inhibition could change tau hyperphosphorylation. Immunocytochemical experiments (Figure 5a,b) found that PFC neurons from P301S Tau mice displayed a significantly higher level of ^S202/T205^p‐Tau, which was remarkably brought down by a short treatment with UNC0642 (1 mg/kg, i.p., 3x, *n* = 10–16 slices, 2–4 mice/group, *F*_1,46(interaction)_ = 76.4, *p* < 0.001, *F*_1,46(genotype)_ = 183.7, *p* < 0.001, *F*_1,46(treatment)_ = 84.1, *p* < 0.001, two‐way ANOVA), while the intensity of NeuN signals was not significantly altered by UNC0642 treatment. Quantitative Western blotting experiments (Figure 5c,d) further confirmed that UNC0642 treatment (1 mg/kg, i.p., 3x) of P301S Tau mice significantly reduced ^S202/T205^p‐Tau (*n* = 4 mice/group, *F*_1,12(interaction)_ = 5.16, *p* < 0.05, *F*_1,12(genotype)_ = 39.92, *p* < 0.001, *F*_1,12(treatment)_ = 5.63, *p* < 0.05, two‐way ANOVA), but not total Tau (*n* = 4 mice/group, *F*_1,12(interaction)_ = 0.28, *p* = 0.61, *F*_1,12(genotype)_ = 103.4, *p* < 0.001, *F*_1,12(treatment)_ = 0.26, *p* = 0.62, two‐way ANOVA). Moreover, co‐immunocytochemical experiments demonstrated the co‐expression of ^S202/T205^p‐Tau with the elevated EHMT2 (Figure 6a,b) and H3K9me2 (Figure 6c,d) in PFC neurons from P301S Tau mice. While low p‐Tau‐positive neurons were found in PFC of WT mice (EHMT2: 7.7%; H3K9me2: 12.3%), most of the p‐Tau‐positive neurons in PFC of P301S Tau mice showed the elevated EHMT2 (71.6%) and H3K9me2 (73.9%). Collectively, it indicates that EHMT inhibition is capable of alleviating the neurofibrillary pathology in PFC of P301S Tau mice.

### 2.4 EHMT inhibition ameliorates transcriptomic dysregulation in PFC of P301S Tau mice

**FIGURE 7:**
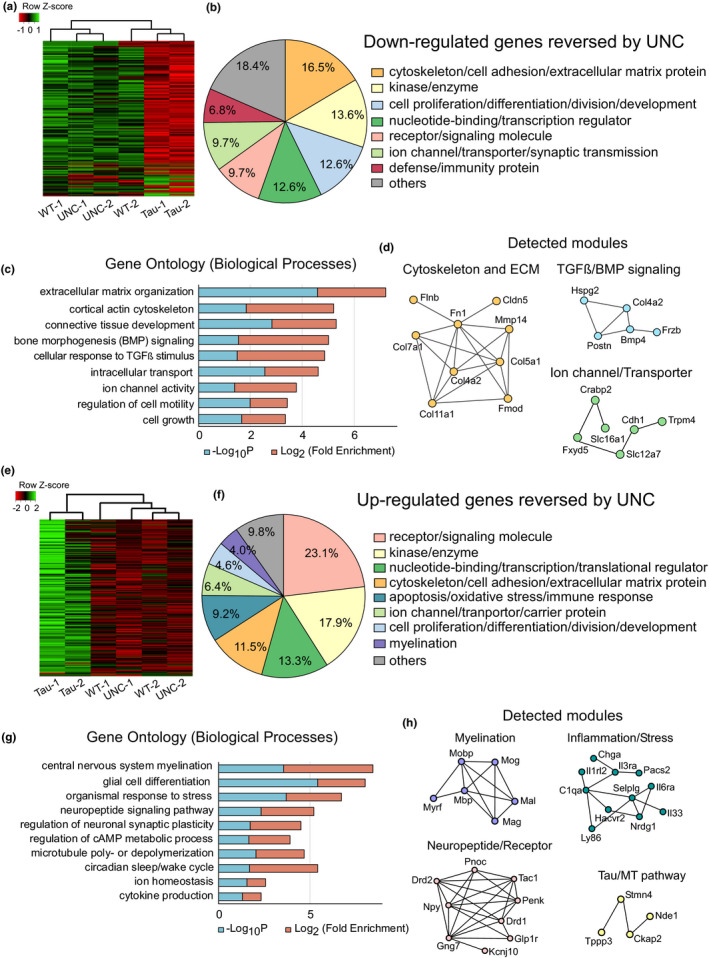
EHMT inhibitor UNC0642 reverses transcriptional changes in PFC of Tau P301S transgenic mice. (a, e) Heat maps representing expression (row *z*‐score) of genes that were down‐regulated (DOWN) (a) or up‐regulated (UP) (e) in saline‐treated P301S Tau samples (Tau) and reversed in UNC0642 (1 mg/kg, i.p., 3x)‐treated P301S Tau samples (UNC). (b, f) Functional protein classification analysis of the DOWN and UNC‐reversed genes (b) or the UP and UNC‐reversed genes (f). (c, g) GO Biological Process analysis of the DOWN and UNC‐reversed genes (c) or the UP and UNC‐reversed genes (g). (d, h) Detected PPI modules in DOWN and UNC‐reversed genes (d) or the UP and UNC‐reversed genes (h)

Given the therapeutic effects of EHMT1/2 inhibitor UNC0642 on cognitive and synaptic impairment in P301S Tau mice, we next examined whether UNC0642 treatment could rescue transcriptional dysregulation in P301S Tau mice caused by the abnormally high level of EHMT2 and repressive H3K9me2. RNA sequencing was used to identify differentially expressed genes (DEGs) in PFC from WT and P301S Tau mice (5–7 months old) with or without UNC0642 treatment. Compared with WT mice, P301S Tau mice exhibited 448 down‐regulated and 610 up‐regulated genes, and UNC0642 treatment significantly reversed 98 of these down‐regulated genes and 173 of these up‐regulated genes (Tables S1 & S2). From the heat maps generated with the expression values of these reversed genes, it is evident that P301S Tau samples clustered separately from WT samples, and UNC0642‐treated Tau samples were closer to WT than non‐treated Tau samples (Figure 7a,e).

Functional protein classification revealed that enzymes, transcription regulators, and receptors were found among UNC0642‐rescued genes in both directions (Tables S3 & S4). Importantly, the down‐regulated genes in P301S Tau mice that were elevated by UNC0642 treatment mainly encode cytoskeletal proteins, extracellular matrix (ECM) molecules, and cellular developmental regulators (Figure 7b). Gene Ontology (GO) analysis revealed that they were enriched in nine cellular process pathways: ECM organization, cortical actin cytoskeleton, tissue development, bone morphogenesis (BMP) signaling, response to TGFβ stimulus, intracellular transport, ion channel activity, regulation of cell motility, and cell growth (Figure 7c). Furthermore, integration of DEGs with the PPI network identified three major functional modules in down‐regulated and reversed genes: cytoskeleton/ECM, TGFβ/BMP signaling, and ion channel (Figure 7d), further supporting the enrichment of these significant pathways.

On the other hand, there are diverse categories of genes up‐regulated in P301S Tau mice and reversed by UNC0642 treatment (Figure 7f). GO analysis revealed 10 enriched cellular processes: central nervous system myelination, glia cell differentiation, multicellular organismal response to stress, neuropeptide signaling pathway, regulation of neuronal synaptic plasticity, regulation of cAMP metabolic process, MT polymerization/depolymerization, circadian sleep/wake cycle, ion homeostasis, and cytokine production (Figure 7g). The PPI network demonstrated four enriched modules in these up‐regulated and reversed genes: myelination, inflammation/stress, neuropeptide/receptor, and Tau/MT pathway (Figure 7H).

## 3 DISCUSSION

Epigenetic dysregulation of gene expression is one of the major contributing factors for cognitive decline related to aging and neurodegenerative diseases (Berson et al., 2018). Histone modification by G9a/GLP (EHMT1/2) is emerging as a pivotal epigenetic mechanism regulating cognitive processes (Maze et al., 2010; Sharma et al., 2017; Yuan et al., 2020). Our current study has provided evidence demonstrating that EHMT2 (G9a), which catalyzes the repressive histone mark H3K9me2 (Barski et al., 2007) at learning and memory genes (Kramer et al., 2011), is highly elevated in PFC neurons of P301S Tau transgenic mice. Moreover, treatment with the EHMT inhibitor UNC0642 ameliorates spatial and recognition memory deficits in P301S Tau mice at the age of 5–7 months old, a late stage that cognitive impairment correlating with neurofibrillary tangle (NFT) deposition has already occurred (DeVos et al., 2017; Mathys et al., 2019; Yoshiyama et al., 2007; Zhang et al., 2014), making it highly promising for clinical usage. The general safety of UNC0642 is suggested by the lack of behavioral abnormalities after treatment (Kim et al., 2017; Kramer et al., 2011; Liu et al., 2013).

Because of the powerful impact of epigenetic drugs on gene expression and the potential side effects with prolonged administration, we have chosen a short treatment paradigm in mice (i.p., once daily for 3 days), which was based on a similar regimen used in human cancer treatment with the FDA‐approved HDAC inhibitor romidepsin (i.v., once a week for 1 month). Similar doses and durations have been used with UNC0642 in prior mouse studies (Kim et al., 2017; Liu et al., 2013; Wang et al., 2020; Zheng et al., 2019). A limitation is the disappearance of behavioral improvement ~7 days after the cessation of UNC0642 treatment, which may be due to the loss of H3K9me2 inhibition. Further studies are needed to examine the effect durations with longer or repeated administrations of UNC0642 at various doses.

Uncovering the physiological basis of UNC0642 treatment is important for understanding the therapeutic effects of UNC0642 on cognitive deficits. Alteration of neuronal activity in cognitive networks has been linked to AD progression (Palop & Mucke, 2016; Rogerson et al., 2014; Styr & Slutsky, 2018). In this study, we have found a remarkable reduction of both intrinsic and synaptic‐driven excitability of PFC principal neurons in P301S Tau mice, as indicated by the lower firing frequency, the longer latency of the first spike, and the higher rheobase current. It is consistent with prior findings on the reduction of neuronal excitability in tauopathies associated with AD in the late phase (Fu et al., 2017; Menkes‐Caspi et al., 2015). More importantly, the diminished excitability of prefrontal cortical neurons is restored by UNC0642 treatment of P301S Tau mice, providing a potential basis for UNC0642‐induced improvement of PFC‐mediated cognitive function.

Changes in excitability are often associated with altered synaptic strength. Our data suggest that hypoactivity in transgenic Tau mice is likely attributable to compromised synaptic excitation in PFC pyramidal neurons. Synaptic dysfunction is an important pathogenic step of neurodegenerative diseases and a perceived basis of cognitive impairment (Mielke et al., 2017). As a major executor of the synaptic pathology, mutant tau can affect pre‐ and postsynaptic protein trafficking and subcellular expression, as well as synaptic organization and transmission (Hoover et al., 2010; Yoshiyama et al., 2007; Zhang et al., 2014). Inhibition of EHMT in P301S Tau mice reverses the defects in glutamatergic synaptic function of PFC, further supporting the association of histone K9 methylation with synaptic dysfunction in AD (Lee et al., 2020; Zheng et al., 2019).

Correlating with cognitive and synaptic impairment, P301S Tau mice also have the pathological tau deposition (DeVos et al., 2017; Mathys et al., 2019; Yoshiyama et al., 2007; Zhang et al., 2014). In this study, we have revealed the remarkable decrease of hyperphosphorylated tau by EHMT inhibition in PFC of P301S Tau mice, which may contribute to the therapeutic effect of UNC0642 on neuronal physiology in mice with tauopathy. The elevated levels of EHMT2+ and H3k9me2+ neurons correlate with neurons with phospho‐tau burden in PFC of P301S Tau mice. The mechanisms underlying the effect of UNC0642 on tau phosphorylation await to be further examined. Given the role of EHMT on transcriptional repression, one possibility is that inhibiting the elevated EHMT in P301S Tau mice led to the disinhibition of genes encoding protein phosphatases that act to remove tau phosphorylation.

Our transcriptomic analysis has provided insights into the potential molecular mechanism for the rescuing effect of EHMT inhibition on synaptic and cognitive deficits in P301S mice. As an epigenetic enzyme mediating the repressive H3K9me2, EHMT1/2 plays an important role in silencing the expression of genes (Tachibana et al., 2005), including those involved in synaptic plasticity and cognition (Kramer et al., 2011; Rodenas‐Ruano et al., 2012; Zheng et al., 2019). Inhibiting EHMT with UNC0642 leads to the restoration of ~22% of the down‐regulated genes (98 out of 448) in P301S Tau mice, which are enriched in cytoskeleton regulation. Cytoskeleton plays a critical role in orchestrating neuronal trafficking and maintaining synaptic function (Guedes‐Dias & Holzbaur, 2019), loss of which contributes directly to memory deficits and correlates directly with AD pathology (Kommaddi et al., 2018). Single‐cell RNAseq of AD human postmortem tissues and an Aβ‐linked familial AD model also found significant loss of cytoskeleton genes in PFC pyramidal neurons (Zhou et al., 2020). Inhibiting EHMT with UNC0642 also leads to the reversal of ~28% of the up‐regulated genes (173 out of 610) in P301S Tau mice, which are enriched in glial cell differentiation and stress responses. Normalization of these altered genes by UNC0642 may collectively help to restore PFC neuronal excitability and synaptic transmission, leading to cognitive improvement.

An important question awaits to be answered is the time line of epigenetic, pathological, physiological, and behavioral changes in AD, which requires longitudinal characterizations. One limitation of our current study is that all experiments are performed at the late stage when all these changes have occurred. It is unknown whether the H3K9me2/EHMT2 levels in PFC increase in an age‐dependent manner in AD humans and Tau AD models, and whether elevation of H3K9me2/EHMT2 is an upstream event prior to NFT formation/synaptic dysfunction/behavioral deficits. One possibility is that H3K9me2/EHMT2 levels start increasing in PFC neurons without NFT pathology at 3 months of age, and those neurons are more susceptible to disease and more easily accumulate NFT pathology at 6 months, which is supported by the significant correlation of p‐Tau and H3K9me2/EHMT2 in P301S Tau mice at the symptomatic stage. Consistently, it has been shown that epigenetic dysregulation occurs early in vulnerable brain regions prior to the onset of clinical symptoms in A and alters the activity of key genes involved in the pathological onset and progression of AD (Bradley‐Whitman & Lovell, 2013; Li et al., 2019).

Overall, our data have not only uncovered an epigenetic mechanism associated with tauopathy, but also provided experimental evidence that targeting EHMT can alleviate synaptic and cognitive deficits in a tau model of neurodegenerative disorders.

## 4 EXPERIMENTAL PROCEDURES

### 4.1 Animals and compounds

Care and experimental manipulation of animals followed the protocol approved by Institutional Animal Care and Use Committee (IACUC) of the University at Buffalo. Transgenic mice (B6C3H/F1 background) carrying the mutant (P301S) human T34 tau isoform (1N4R; Jackson Labs) were obtained as previously described (Yoshiyama et al., 2007). Mouse tail was used for PCR Genotyping. Both male and female wild‐type (WT) and P301S Tau transgenic littermates (5–7 months old) were used in this study. Animals were housed with 2–4 gender‐matched conspecifics of either genotype in a 12‐h light‐dark cycle with food and water ad libitum. UNC0642 (Tocris) was dissolved in DMSO and then diluted with saline before use. DMSO concentration in the final solution was 0.18%. Mice were treated with UNC0642 (1 mg/kg, i.p., once daily for three consecutive days) or vehicle (saline containing 0.18% DMSO), and experiments were carried out 24 h later. Mice with different genotypes were randomly allocated to UNC0642‐ or vehicle‐treated groups. Investigators were blind to all experimental groups.

### 4.2 Immunohistochemistry

Whole brains were immediately removed after mice were transcardially perfused with PBS followed by 4% paraformaldehyde, post‐fixed at 4°C for 24 h, and coronally cut into 40 μm slices. After washing with PBS, slices were incubated in the primary antibodies against EHMT2 (1:100, Abcam, ab185050), H3K9me2 (1:50, CST, D85B4), NeuN (1:500, Millipore, MAB377, or 1:500, Novus Biologicals, NBP1‐77686), or P‐Tau (1:500, Thermo Fisher, MN1020) for 12–16 h at 4°C. After three rinses in PBS, slices were incubated with corresponding secondary antibodies (Alexa Fluor 488, 1:1000, Thermo Fisher, A27034; Alexa Fluor 594, 1:1000, Thermo Fisher, A‐11032) for 1 h at room temperature, followed by three rinses with PBS. Slices were mounted onto slides with anti‐fading mounting media (VECTASHIELD, Vector Laboratories). Images were taken by a confocal microscope (Leica TCS SP8). All specimens were imaged and analyzed with identical parameters.

Analyses of immunostaining signals were performed with Image J (NIH). Each data point in the graph represented the average fluorescent signal intensity in each region of interest (ROI) normalized to WT controls. About 10–26 neurons were assessed in each ROI. About 3–4 ROIs in PFC of each mouse and 2–4 mice per group were evaluated.

### 4.3 Western blotting

PFC punches were collected from brain slices and homogenized in 1% SDS buffer. SDS electrophoresis and transferring were performed to detect target proteins by incubating overnight with the following primary antibodies: Phospho‐Tau (AT8) (1:500, Thermo Fischer, MN1020), Tau (Tau‐5) (:500, Thermo Fischer, AHB0042), or GAPDH (1:2000, Cell Signaling, 5174). After secondary antibodies (horseradish peroxidase‐conjugated) incubation, enhanced chemiluminescent (ECL) reaction was performed using ECL substrate (SuperSignal™ West Femto Maximum Sensitivity Substrate or SuperSignal™ West Pico PLUS Chemiluminescent Substrate, Thermo Scientific). Luminescence was detected by Chemidoc XRS system (Bio‐Rad), and density of blots was quantified by ImageJ software (NIH).

### 4.4 Behavioral testing

Behavioral tests were performed in dimly lit rooms and scored by trained operators blind to experimental conditions. Any‐maze behavior tracking system (Stoelting) was used for tracing animals and generating heat maps illustrating time in different locations of the arena. Mice were habituated to the behavioral testing room in their home cages for at least 30 min. Between trials, animals were returned to the home cages. To mask olfactory cues, all testing apparatuses were cleaned using 75% ethanol. In some experiments, the same mice before and after UNC0642 treatment were monitored in behavioral tests. The timeline of drug treatment and behavioral tests are illustrated in Figure S1.

#### 4.4.1 Barnes maze

A round platform (36‐inch diameter) with eight equally spaced holes at the edge was used. One of the holes had an escape box attached during the training phases. Visual cues were placed on sidewalls to indicate the hole locations. An overhead light brightly illuminated the apparatus as an aversive stimulus to force the animal to run into the escape box. After 5‐min habituation, the animal was allowed to explore the platform till entering the escape box during the two training phases (5‐min interval). After a 15‐min break in the home cage, the animal was positioned on the apparatus (escape box removed) to carry out the test phase (5 min) using distal visual cues. The time spent around the correct hole (T1) and all the other incorrect holes (T2) was recorded. The spatial memory index (T1/T2) was calculated as previously described (Cao et al., 2020; Wang et al., 2018). For repeated measurements, visual cues were changed before training.

#### 4.4.2 Novel object recognition

After habituation on a circular platform (24‐inch diameter) for 5 min, the test animal was allowed to explore two identical objects on the platform for 5 min. After a 5‐min interval, the mouse was returned to explore the platform containing one original familiar object and a novel object for 5 min. The amount of time spent interacting with each object was scored. The discrimination ratio was calculated by (*T*_Nov_ − *T*_Fam_)/(*T*_Nov_ + *T*_Fam_), where *T*_Nov_ and *T*_Fam_ indicate the time spent with the novel and familiar object, respectively. All the familiar and novel objects used in each repeated measurement were unique with no repeat.

### 4.5 Whole‐cell recording in brain slices

#### 4.5.1 Slice preparation

Mouse was decapitated under 1–3% isoflurane (Sigma) anesthesia, and the brain was quickly removed and coronally cut into 300‐µm slices with a vibratome (Leica VP1000S, Leica Microsystems Inc.) in an ice‐cold sucrose solution. The slices were recovered for at least 1 h at 35°C in standard artificial cerebrospinal fluid (ACSF) and maintained at room temperature afterward.

#### 4.5.2 Electrophysiological recordings

The slice was transferred into a recording chamber on an upright microscope (Olympus) and perfused with oxygenated ACSF at room temperature (22°C). Neurons were viewed under a water‐immersion lens (40×) and a CCD camera. A Multiclamp 700 A amplifier with Clampex 8.2 software and Digidata1322A (Molecular Devices) were used for recordings. A pipette puller (Model P‐97, Sutter Instrument Co.) was used to pull recording pipettes from glass capillaries (1.5 mm OD and 0.86 mm ID) with resistance at 3–5 MΩ. Whole‐cell voltage‐clamp recording was used to measure synaptic currents in PFC layer V pyramidal neurons (Tan et al., 2019; Wang et al., 2018). Pipette was filled with the intracellular solution (in mM: 130 Cs‐methanesulfonate, 10 CsCl, 4 NaCl, 1 MgCl_2_, 10 HEPES, 5 EGTA, 2 QX‐314, 12 phosphocreatine, 5 MgATP, 0.2 Na_3_GTP, 0.1 leupeptin, pH 7.3, 270 mOsm). A stimulating electrode (FHC) was placed 100 µm away from the recorded neuron. Excitatory postsynaptic currents (EPSC) were elicited by a series of pulses from an S48 stimulator (Grass Technologies) with different intensities that delivered at 0.05 Hz. To measure NMDAR‐EPSC, CNQX (25 μM) and bicuculline (20 μM) were added, and the neuron was depolarized to +40 mV for 3 s (removing Mg^2+^ block) before stimulation. To record evoked and spontaneous AMPAR‐EPSC, bicuculline (20 μM) and D‐APV (25 μM) were added, and the cell was held at −70 mV. For PPRs, two pulses with different intervals (20–300 ms) were used to evoke synaptic currents.

Current‐clamp recording was performed to record action potential (AP) with intracellular solution (in mM: 100 K‐gluconate, 20 KCl, 10 HEPES, 4 ATP, 0.5 GTP, and 10 phosphocreatine, pH 7.3, 265–270 mOsm). To elevate basal neuronal activity, slices were perfused in a modified ACSF with low MgCl_2_ (0.5 mM; Tan et al., 2021). For spontaneous AP (sAP) recording, a small current was injected to adjust the membrane potential to −60 mV. Evoked AP (eAP) was obtained with incremental steps of current injections (0–120 pA).

### 4.6 RNA sequencing and bioinformatic analysis

RNA extraction from PFC samples of biological duplicates in each group used the RNAeasy Mini kit (Qiagen), combined with the RNase‐free DNase step (Qiagen). The strand‐specific RNA sequencing library was generated from the purified RNA (1 μg) by using TruSeq stranded total RNA and Ribo‐zero kits (Illumina). The sequencing was analyzed by Illumina HiSeq 2500 platform at the Genomics and Bioinformatics Core of University at Buffalo. Reads were trimmed using Cutadapt to remove the 3′ end adapters and trailing sequences, then aligned to mouse RefSeq mRNAs using TopHat2 with default parameters. Reads count for each gene was estimated with featureCounts. Differences in gene expression levels between samples were defined with at least 1.2‐Fold Change (FC) and *p* < 0.05. GO annotation was carried out as we previously described (Cao et al., 2020).

Cytoscape software was applied to visualize the gene interaction relationship network and identify Hub Genes and Key Pathways. We utilized the CytoHubba application in Cytoscape, employing MCC (Maximal Clique Centrality) algorithm to rank top genes. MCODE plugin was used to present dense regions of protein/gene interaction networks. STRING plugin was used for protein–protein interaction (PPI) network analysis.

### 4.7 Quantitative real‐time PCR

Total RNA was extracted from PFC punches with Trizol (Invitrogen), then incubated in DNase I (Invitrogen) to remove any contaminating DNA. The mRNA was converted into cDNA by using an iScript reverse transcription kit (Bio‐Rad). The iCycler iQ™ Real‐Time PCR Detection System and iQ™ Supermix (Bio‐Rad) were used for qPCR. Fold changes in the target genes were determined by the following formula: 2^−Δ(ΔCT)^, where Δ(ΔCT) = ΔCt(P301S) − ΔCt(WT), and ΔCt = Ct(target gene) − Ct(GAPDH). Ct (threshold cycle) is defined as the fractional cycle number at which the fluorescence reaches 10× the standard deviation of the baseline. The total reaction mixture (18 μl) was amplified in a 96‐well thin‐wall PCR plate (Bio‐Rad). PCR cycling parameters: 95°C for 5 min, followed by 40 cycles of 95°C for 30 s, 55°C for 30 s, and 72°C for 60 s. Primers for EHMT1 (Forward ‐ CATAGCAAAAGCAGACACAA; Reverse ‐ ACTTTCCAAGGTTTCCTTTC) and EHMT2 (Forward ‐ GCTCCACCTGTCTACATCAT; Reverse ‐ GCAGATGTTTTCCTCATTGT).

### 4.8 Statistical analyses

Clampfit (Molecular Devices) and Mini Analysis (Synaptosoft) were used for electrophysiological data analyses. GraphPad Prism 8.0 was used for all statistical analyses. Student's *t* test (two‐tailed paired or unpaired) was used for statistical analyses of experiments with two groups. ANOVA (one‐way, two‐way or two‐way repeated measures) with Bonferroni post hoc test was used for statistical analyses of experiments with more than two groups. All data are presented as Mean ± SEM.

## CONFLICT OF INTEREST

The authors report no competing financial or other interests.

## AUTHOR CONTRIBUTIONS

W.W. designed experiments, performed electrophysiological and behavioral experiments, and wrote the draft. Q.C. performed immunohistochemical, biochemical and behavioral experiments, and parts of bioinformatic analysis. T.T. performed parts of electrophysiological analysis. F.Y. and J.W. performed parts of bioinformatic analysis. Z.Y. designed experiments, supervised the project, and wrote the paper.

## Supporting information

Fig S1

Tables S1‐S4

## Data Availability

Genomic data have been deposited in the GEO public repository (GSE182170).
