# Supplementary figures and images for: Epigenetic treatment of behavioral and physiological deficits in a tauopathy mouse model
Source: Aging Cell. 2021 Sep 21;20(10):e13456. doi: 10.1111/acel.13456 (PMC8520711; doi:10.1111/acel.13456)

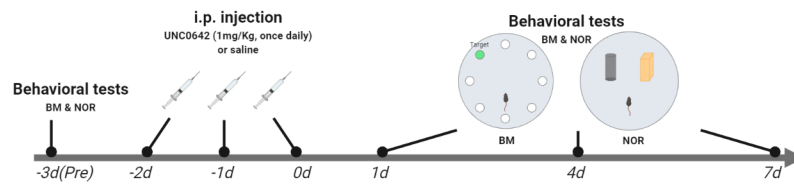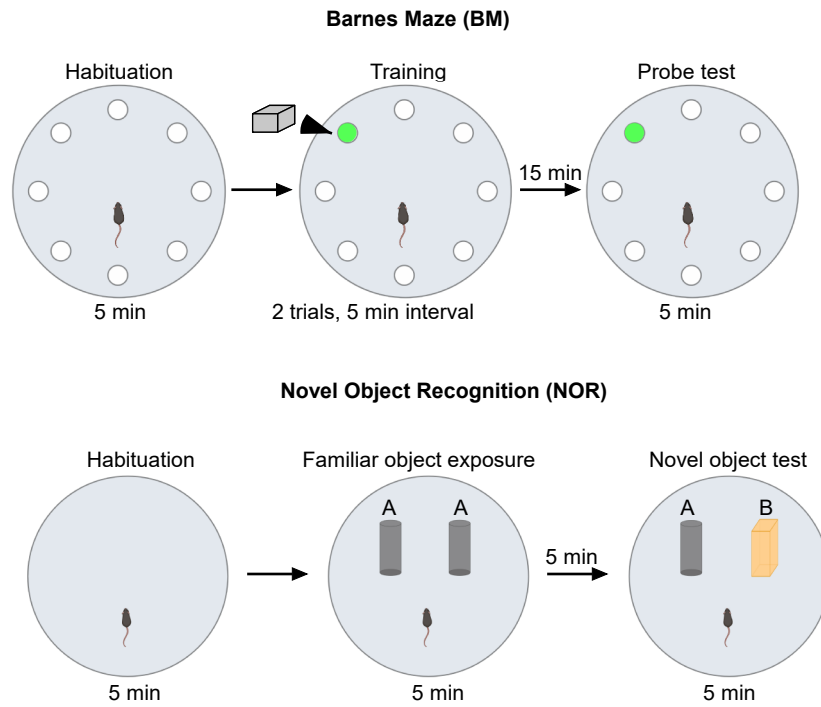

**Supplementary Figure 1** Diagrams showing the time line of treatment and behavioral tests.

Supplement: Supplementary file 1 — Fig S1 [file ACEL-20-e13456-s002.pdf]
