# Supplementary material for: Epigenetic treatment of behavioral and physiological deficits in a tauopathy mouse model
Source: Aging Cell. 2021 Sep 21;20(10):e13456. doi: 10.1111/acel.13456 (PMC8520711; doi:10.1111/acel.13456)
Supplement: Supplementary file 2 — Tables S1‐S4 [file ACEL-20-e13456-s001.pdf]

Suppl. Table 1 List of genes down-regulated in P301S Tau transgenic mice and reversed by UNC0642 treatment

Gene Symbol	log FC (Tau vs WT)	Fold Change (Tau vs WT)	FDR (Tau vs WT)	AveExpr (Tau vs WT)	P value (Tau vs WT)	log FC (UNC vs Tau)	Fold Change (UNC vs Tau)	FDR (UNC vs Tau)	AveExpr (UNC vs Tau)	P value (UNC vs Tau)	Description
Frzb	-1.36501179	-2.575784308	1.74005E-05	5.817676227	1.7227E-18	0.992213481	1.98923467	4.6396E-07	5.817676227	9.8575E-12	Secreted frizzled-related protein 3
Hhipl1	-1.233571908	-2.35148464	0.000217249	4.70538236	8.97167E-14	0.809560312	1.752677201	1.4118E-05	4.70538236	1.83599E-07	HHIP-like protein 1
Btg2	-1.072127793	-2.102532051	1.16348E-06	4.880250811	2.07733E-09	0.374566464	1.296449907	7.0625E-08	4.880250811	0.024001685	Protein BTG2
Ccdc85c	-0.643321207	-1.561920699	0.019038258	5.039910417	9.98641E-09	0.305054834	1.235465594	0.44939029	5.039910417	0.003345726	Coiled-coil domain-containing protein 85C
Cldn5	-1.104305483	-2.149953538	0.002800435	4.627496825	1.55728E-08	0.616213123	1.532846384	0.00041497	4.627496825	0.000742243	Claudin-5
Leng8	-0.712572288	-1.638723315	4.99385E-10	4.435718954	5.35955E-07	0.310952122	1.240526129	0.00017033	4.435718954	0.018065734	Leukocyte receptor cluster member 8 homolog
Hspg2	-1.297539397	-2.458092824	0.043459033	3.1748285	1.8914E-06	0.570405076	1.48494045	0.43393974	3.1748285	0.027939672	heparan sulfate proteoglycan core protein
Snhg11	-0.612842094	-1.529268887	1.9178E-14	4.977841456	1.17151E-05	0.492008914	1.406401889	3.1354E-08	4.977841456	0.000136098	Small nucleolar RNA host gene 11
Olfml2a	-1.351957909	-2.552583065	0.058267406	3.745206156	1.76566E-05	0.712963746	1.639168024	0.48904196	3.745206156	0.017538593	Olfactomedin-like protein 2A
Pla2g4e	-0.849167411	-1.801460989	0.159655038	3.094170503	0.000100899	0.422142902	1.339916317	0.27201598	3.094170503	0.036149024	Cytosolic phospholipase A2 epsilon
Adgrg3	-1.809456601	-3.50510242	0.148176089	1.450586496	0.000163253	1.25314578	2.383605994	0.33204799	1.450586496	0.007764537	Adhesion G protein-coupled receptor G3
Il13ra2	-2.705083717	-6.52095705	0.344623309	1.142380073	0.000172091	1.839695297	3.579344232	0.43533802	1.142380073	0.010735545	Interleukin-13 receptor subunit alpha-2
Slc9a2	-1.111042382	-2.160016573	0.087669521	2.961498467	0.000191302	0.567399064	1.481849636	0.09035911	2.961498467	0.045078949	Sodium/hydrogen exchanger
Spaca6	-0.716372216	-1.643045253	0.00372624	9.203428138	0.000197953	0.627336589	1.544710614	0.0244372	9.203428138	0.000462994	Sperm acrosome membrane-associated protein 6
Vstm4	-1.297856236	-2.45863272	0.117695651	3.101087451	0.000275082	0.89901558	1.864793111	0.38819741	3.101087451	0.007935629	V-set and transmembrane domain-containing protein 4
Grrp1	-2.081106295	-4.231315597	0.440329337	2.683799624	0.000350477	1.807794913	3.501067587	0.34751142	2.683799624	0.001253052	Protein FAM110D
Col11a1	-1.107810983	-2.155183906	0.067469674	2.687212239	0.000394867	0.616429578	1.533076382	0.17219031	2.687212239	0.036037268	Collagen alpha-1(XI) chain
Crabp2	-1.904606779	-3.744068368	0.069947248	1.217495364	0.000405695	1.189592669	2.280883355	0.42365061	1.217495364	0.022115353	Cellular retinoic acid-binding protein 2
Col23a1	-0.901840979	-1.868448732	0.000361607	5.57391498	0.000422563	0.508739114	1.422806149	0.33575423	5.57391498	0.033112101	Collagen alpha-1(XXIII) chain
Stx19	-2.096385006	-4.27636503	0.393885562	1.147484578	0.000433455	1.469077792	2.768448706	0.366906	1.147484578	0.01243776	Syntaxin-19
Akap13	-0.401795067	-1.321150725	0.001002665	2.393736914	0.000499031	0.317760999	1.246394696	0.40263871	2.393736914	0.002885456	A-kinase anchor protein 13
Trpm4	-0.623122653	-1.540205286	0.09269247	4.740775121	0.000701355	0.344779669	1.269957017	0.23001714	4.740775121	0.044632081	Transient receptor potential cation channel member 4
Col7a1	-1.599399151	-3.030170874	0.324402997	4.995613605	0.000708552	0.892891536	1.856894095	0.28623469	4.995613605	0.048897302	Collagen alpha-1(VII) chain
Zfp386	-0.533804337	-1.447741812	0.201964558	3.843931662	0.00087808	0.352425508	1.276705264	0.06433358	3.843931662	0.017585561	Zinc finger protein 386 (Kruppel-like)
Fmod	-1.043232924	-2.060840605	0.067469674	5.181668868	0.000878788	0.833185463	1.781614819	0.33072331	5.181668868	0.004532365	Fibromodulin
Postn	-1.163729595	-2.240358478	0.028101583	4.244942375	0.000912159	0.643159199	1.561745312	0.0563741	4.244942375	0.04985345	Periostin
Col5a1	-0.835350064	-1.784289937	0.313912835	5.238749164	0.000928038	0.535994245	1.449941049	0.31608015	5.238749164	0.022644693	Collagen alpha-1(V) chain
Lrrc29	-1.298847318	-2.460322296	0.264643503	2.690007733	0.00094724	0.805380876	1.747607105	0.44937953	2.690007733	0.031089841	Leucine rich repeat containing 29, isoform CRA_c
Hmcn1	-0.839343498	-1.789235761	0.233984583	7.31273167	0.000989886	0.497694878	1.411955752	0.45418146	7.31273167	0.036951245	Hemicentin-1
Mmp14	-0.898552183	-1.864194232	0.282199576	5.991813894	0.00110696	0.751726736	1.683806944	0.28206795	5.991813894	0.003573129	Matrix metalloproteinase-14
Cd93	-0.791324247	-1.730662306	0.199157598	4.81911336	0.001486796	0.755002811	1.687634881	0.4316684	4.81911336	0.001169652	Complement component C1q receptor
Wdr90	-0.490499253	-1.404930976	0.201730278	5.342959343	0.001547866	0.303473777	1.234112383	0.23524293	5.342959343	0.035632588	WD repeat-containing protein 90
Rab40b	-0.510134724	-1.424183185	0.152759314	6.217579044	0.001681911	0.398757061	1.318371592	0.14777602	6.217579044	0.008061096	Ras-related protein Rab-40B
Ptgfr	-1.818895456	-3.528109788	0.124271083	2.737597859	0.001912373	1.418302091	2.672707746	5.8571E-12	2.737597859	0.011191708	Prostaglandin F2-alpha receptor
Malt1	-0.680834418	-1.603066659	0.130178697	6.755859738	0.00248448	0.468466155	1.383637629	0.23001714	6.755859738	0.025927622	lymphoma translocation protein 1 homolog
Etnppl	-0.801247676	-1.742607522	0.38699176	3.825979007	0.00277799	1.979935678	3.944754937	0.2768335	3.825979007	1.05225E-15	Ethanolamine-phosphate phospho-lyase
Wtip	-0.957252714	-1.941609018	0.042974802	0.350653177	0.003850627	0.742748592	1.673360855	0.36467485	0.350653177	0.017731256	Wilms tumor protein 1-interacting protein
Bmp4	-1.381878095	-2.606074082	0.101184435	0.410066601	0.003885833	0.947899828	1.929062423	0.27152851	0.410066601	0.039095448	Bone morphogenetic protein 4
Chst9	-1.898145213	-3.727336869	0.431248816	0.102988259	0.00394955	1.627198793	3.089126163	0.34683997	0.102988259	0.010586723	Carbohydrate sulfotransferase 9
Spata20	-2.651103891	-6.281477275	0.02639729	-0.736748343	0.004042923	2.249129558	4.75395932	0.2652907	-0.736748343	0.011848796	Spermatogenesis-associated protein 20
Slc16a1	-0.368795453	-1.291274259	0.069338113	3.319823992	0.004198909	0.349028055	1.273702244	0.366906	3.319823992	0.00333144	Monocarboxylate transporter 1
Amn1	-0.431549354	-1.348681189	0.075993557	2.642542359	0.004361608	0.346370011	1.271357715	0.15290192	2.642542359	0.013645517	Antagonist of mitotic exit network 1
L3hypdh	-1.012401229	-2.017265856	0.392542334	0.866077905	0.004401072	0.753119416	1.685433161	0.36657563	0.866077905	0.026047145	Trans-L-3-hydroxyproline dehydratase
Otud7a	-0.350859936	-1.275320571	0.383690305	7.450324183	0.004540409	0.353306489	1.277485121	0.22228024	7.450324183	0.001981341	OTU domain-containing protein 7A
Slc12a7	-0.933585667	-1.910017249	0.333919721	1.662830187	0.004546785	0.674441911	1.595979268	0.19162759	1.662830187	0.029869715	Solute carrier family 12 member 7
Fam228a	-1.137214014	-2.199558564	0.237231965	0.803748975	0.005069733	0.790006026	1.729081685	0.23476762	0.803748975	0.042233299	Protein FAM228A
Gpr171	-1.044502078	-2.062654345	0.264643503	0.924024525	0.005258184	0.710147924	1.63597185	0.34599644	0.924024525	0.047453035	Probable G-protein coupled receptor 171
Fam181a	-1.461383513	-2.753723131	0.090499246	-1.046457915	0.00550541	1.201433329	2.299680326	0.45000782	-1.046457915	0.016525771	Family with sequence similarity 181, member A
C1rl	-2.025411379	-4.071079455	0.33463598	0.032984824	0.006115662	1.572235046	2.973650401	0.47392637	0.032984824	0.030590187	Complement C1r subcomponent-like protein
Flnb	-0.355964436	-1.279840864	0.047686417	6.058429524	0.006793093	0.270670456	1.206368326	0.13875743	6.058429524	0.025501592	Filamin-B
Rnpc3	-0.417605018	-1.335708338	0.328962918	5.448804417	0.007103385	0.294510492	1.226468765	0.14392357	5.448804417	0.040281019	RNA-binding protein 40
Tbkbp1	-0.3827765	-1.303848729	0.316090096	6.708197218	0.007306784	0.282169339	1.216022008	0.46728848	6.708197218	0.032784696	TANK-binding kinase 1-binding protein 1
Bloc1s6	-0.362304527	-1.28547765	0.158278841	5.783708694	0.007511089	0.329441404	1.256526766	0.11701476	5.783708694	0.008547565	lysosome-related organelles complex 1 subunit 6
Evc2	-0.919017881	-1.890827668	2.72069E-06	4.868283422	0.007547025	1.106389735	2.153061807	0.37660149	4.868283422	0.000604061	Limbin
Prrx2	-2.179369541	-4.529555688	0.385182069	0.38809606	0.007685455	2.142583373	4.415520074	0.2184211	0.38809606	0.006314812	Paired mesoderm homeobox protein 2
Zfp984	-1.377314596	-2.597843633	0.199157598	-0.16584722	0.00775333	2.945311196	7.702416812	0.40069209	-0.16584722	3.63369E-11	Zinc finger protein 984
Reck	-0.633296103	-1.551104733	0.352674908	3.545066514	0.008416391	0.515851586	1.429837888	0.17219031	3.545066514	0.021721399	Reversion-inducing protein with Kazal motifs
Miip	-0.648455476	-1.567489171	0.034406465	1.630608895	0.009085746	0.793195028	1.732907956	0.22847278	1.630608895	0.000612563	Migration and invasion-inhibitory protein
Fxyd5	-0.710286773	-1.636129308	0.269691564	1.6562293	0.009143098	0.597791091	1.51339763	0.45288501	1.6562293	0.019344197	FXYD domain-containing ion transport regulator 5

Clba1	-0.45299646	-1.368880455	0.089179063	4.137649203	0.009848166	0.515965993	1.42995128	0.44838307	4.137649203	0.001545684	Uncharacterized protein C14orf79 homolog
Gbp11	-1.540780116	-2.909517888	0.165267373	0.627345326	0.01000006	1.330453431	2.514817018	0.47527376	0.627345326	0.019541177	Guanylate binding protein 11
Snrnp70	-0.365083663	-1.287956317	0.068844793	2.456266222	0.010476756	0.272467977	1.207872334	0.50846865	2.456266222	0.037744773	U1 small nuclear ribonucleoprotein 70 kDa
Tspan18	-1.188162242	-2.278622989	0.158278841	1.62409376	0.010784665	1.155369668	2.227413903	0.41617597	1.62409376	0.008483691	Tetraspanin-18
Col4a2	-0.379398008	-1.300798959	0.196744725	4.868931543	0.011840445	0.312001975	1.241429191	0.46686981	4.868931543	0.025043839	Collagen alpha-2(IV) chain
Hars2	-0.368171039	-1.290715502	0.001090859	-0.557384483	0.012809212	0.293596938	1.225692376	0.16077944	-0.557384483	0.032243522	D-tyrosyl-tRNA(Tyr) deacylase 1
Tnip2	-0.453719606	-1.369566773	0.027657974	1.977016587	0.013722041	0.393162665	1.313269189	0.49007949	1.977016587	0.021745031	TNFAIP3-interacting protein 2
Notch3	-0.620435849	-1.537339553	0.264291735	3.553427469	0.013830987	0.493199625	1.407563125	0.36115312	3.553427469	0.036060262	Neurogenic locus notch homolog protein 3
Haus4	-1.052431545	-2.0740225	0.218715216	2.202883419	0.013893695	0.945095355	1.925316135	0.34705564	2.202883419	0.019160728	HAUS augmin-like complex subunit 4
Rnasel	-0.382545886	-1.303640327	0.255552956	4.59911553	0.013977651	0.287581034	1.220591995	0.42911058	4.59911553	0.046916147	2-5A-dependent ribonuclease
Nxf7	-0.937654558	-1.915411751	0.025597363	0.099082165	0.01431743	1.060027693	2.084971543	0.22657591	0.099082165	0.003110242	Nuclear RNA export factor 7
Sphk1	-0.845771762	-1.797225905	0.389146076	2.868415423	0.01470107	0.677353988	1.599204003	0.38819741	2.868415423	0.037467514	Sphingosine kinase 1
Wsb1	-0.330019992	-1.257030793	0.071557861	2.947562156	0.016125286	0.317580023	1.246238354	0.4519781	2.947562156	0.012062955	WD repeat and SOCS box-containing protein 1
Cdh1	-1.081423053	-2.116122364	0.21128498	2.698008437	0.017874696	1.146809357	2.214236552	0.36115312	2.698008437	0.007466604	Cadherin-1
Smpd4	-0.334095134	-1.260586514	0.385805732	3.458850545	0.01862326	0.300664845	1.231711899	0.49883939	3.458850545	0.021709523	Sphingomyelin phosphodiesterase 4
Fn1	-0.558994928	-1.473242506	1.73364E-05	4.027148145	0.020570349	0.539654913	1.453624775	0.07185239	4.027148145	0.015701084	Fibronectin
Grin2c	-0.302106428	-1.232943277	0.226517994	3.975979851	0.022183998	0.304471544	1.234966189	0.10306694	3.975979851	0.012713202	Glutamate receptor ionotropic, NMDA 2C
BC005561	-0.363994116	-1.286983999	0.388562681	5.734031283	0.022809435	0.436031537	1.352877804	0.32113985	5.734031283	0.003173616	CDNA sequence BC005561
Abra	-1.020578709	-2.028732583	0.170192592	0.841212576	0.023071918	1.039618388	2.055683826	0.49883939	0.841212576	0.013782688	Actin-binding Rho-activating protein
Zscan30	-0.997026033	-1.995881453	0.336699542	0.818429907	0.023667566	0.906928711	1.875049537	0.10152109	0.818429907	0.029889996	Zinc finger and SCAN domain-containing 30
Perm1	-1.225520463	-2.338397939	0.266700699	0.851007786	0.023884249	1.047159025	2.066456543	0.14278256	0.851007786	0.041895867	PGC-1 and ERR-induced regulator in muscle protein 1
Adam8	-0.888580487	-1.851353623	0.176084762	0.326083261	0.024294649	1.165531719	2.243158741	0.32355988	0.326083261	0.001477334	Disintegrin domain-containing protein 8
Ing4	-0.796731642	-1.737161208	0.040017337	-0.01852489	0.0256939	0.715551716	1.642111075	0.09330368	-0.01852489	0.033329469	Inhibitor of growth protein 4
Spag5	-0.454384744	-1.370198341	0.29916803	5.657452007	0.027197072	0.542290141	1.456282393	0.36115312	5.657452007	0.004494881	Sperm-associated antigen 5
Clcn1	-0.778699998	-1.715584275	0.218708009	2.766620929	0.027577495	0.83484954	1.783671008	0.06433358	2.766620929	0.011413162	Chloride channel protein 1
Luc7l3	-0.289687737	-1.222375673	0.042472093	2.371034064	0.033015376	0.334617817	1.261043303	0.44937953	2.371034064	0.007497528	Luc7-like protein 3
Chst14	-0.922769359	-1.895750835	0.228035575	-0.155252445	0.033331228	1.089560891	2.128092545	0.34751142	-0.155252445	0.007180968	Carbohydrate sulfotransferase 14
Kif26a	-0.476074365	-1.390953667	0.21128498	6.415888222	0.033546818	0.411990056	1.33051987	0.47699728	6.415888222	0.047448629	Kinesin-like protein KIF26A
Alg6	-0.365720198	-1.288524705	0.058646543	0.711144412	0.033847371	0.403736304	1.322929612	0.50342855	0.711144412	0.0115947	Dolichyl pyrophosphate alpha-1,3-glucosyltransferase
Cspp1	-0.33773109	-1.263767517	1.17025E-05	6.076533014	0.034641676	0.354551474	1.278588014	0.14809658	6.076533014	0.016255316	Centrosome and spindle pole associated protein 1
Zbtb49	-0.443556629	-1.359952846	0.264897585	4.06401631	0.034825665	0.530785336	1.444715417	0.49692015	4.06401631	0.006485963	Zinc finger and BTB domain-containing protein 49
Actl10	-0.785599825	-1.723808874	0.155786751	1.08068605	0.035841552	0.799233727	1.740176604	0.38948266	1.08068605	0.022592869	Actin-like protein 10
Mab21l1	-0.884646863	-1.846312634	0.04349203	-0.429857199	0.035893837	1.058042842	2.082105024	0.28549146	-0.429857199	0.007215382	Putative nucleotidyltransferase MAB21L1
Ccdc73	-0.417168762	-1.335304494	0.266700699	8.985928566	0.040038938	0.555731095	1.469913333	0.34982175	8.985928566	0.00318681	Coiled-coil domain-containing protein 73
Mamstr	-0.685681236	-1.608461312	0.14829128	0.404438542	0.040238561	0.833911291	1.782511385	0.46129314	0.404438542	0.007450724	MEF2-activating motif transcriptional regulator
Ppp1r42	-1.02936692	-2.041128371	0.329926939	0.705661651	0.045085617	1.128870141	2.186874062	0.29693693	0.705661651	0.019302774	Protein phosphatase 1 regulatory subunit 42
Pdss1	-0.369298993	-1.291725028	0.295911299	1.394852111	0.04738205	0.343512289	1.268841874	0.22209082	1.394852111	0.047113477	Decaprenyl-diphosphate synthase subunit 1
Nsun5	-0.452762847	-1.368658812	0.355296168	1.831424424	0.048438669	0.44530167	1.3615988	0.2741252	1.831424424	0.03701473	Probable 28S rRNA (cytosine-C(5))-methyltransferase
Setd4	-0.477132894	-1.391974608	0.435394694	3.501310185	0.049281012	0.527273896	1.441203335	0.49785551	3.501310185	0.019456454	SET domain-containing protein 4

Suppl. Table 2 List of genes up-regulated in P301S Tau transgenic mice and reversed by UNC0642 treatment

Gene Symbol	log FC (Tau vs WT)	Fold Change (Tau vs WT)	FDR (Tau vs WT)	AveExpr (Tau vs WT)	P value (Tau vs WT)	log FC (UNC vs Tau)	Fold Change (UNC vs Tau)	FDR (UNC vs Tau)	AveExpr (UNC vs Tau)	P value (UNC vs Tau)	Description
Rpl3	1.860826883	3.632157801	3.45055E-10	3.52611666	4.6493E-14	-1.79528457	-3.47083929	1.2521E-12	3.526116664	1.68709E-16	60S ribosomal protein L3
Plin4	2.27704129	4.846829357	3.06405E-09	3.36147531	6.8809E-13	-0.72209284	-1.64957325	0.215462038	3.361475308	0.007044975	Perilipin-4
Per2	1.172835786	2.254544188	4.26759E-08	5.37769653	1.9895E-11	-0.55281387	-1.46694407	0.048360119	5.37769653	0.000380104	Period circadian protein homolog 2
Ighm	1.993841987	3.982962749	8.06277E-06	4.52430744	6.5183E-09	-2.07946305	-4.22649882	1.98908E-08	4.524307444	5.3602E-12	Ig mu chain C region
Per1	0.742372613	1.67292482	0.000124218	5.69058674	1.5621E-07	-0.47042465	-1.38551723	0.033057368	5.690586743	0.000225323	Period circadian protein homolog 1
Cthrc1	1.810709814	3.508148489	0.087669521	7.5177707	9.7306E-07	-2.12042011	-4.34820544	0.212289847	7.517770701	4.45525E-10	Collagen triple helix repeat-containing protein 1
Sst	0.609107215	1.525315004	0.001053288	6.67851397	2.0342E-06	-0.45051387	-1.36652691	0.021723766	6.678513974	0.000101033	Somatostatin
Mt1	0.565877854	1.480287964	0.0032198	6.34956864	9.2552E-06	-0.36527491	-1.28812707	0.103453895	6.349568641	0.001566785	Metallothionein-1
Haus2	0.871990559	1.83018636	0.003529181	4.34031142	1.0937E-05	-0.53170193	-1.44563359	0.138490982	4.340311418	0.002848815	HAUS augmin-like complex subunit 2
Acaa1b	2.310189985	4.95948386	0.34183186	4.9189724	4.3651E-05	-1.40871128	-2.65499893	0.032609684	4.9189724	0.002999838	3-ketoacyl-CoA thiolase B, peroxisomal
Mt2	0.975250033	1.965981891	0.017300602	2.5428004	8.5538E-05	-0.73931348	-1.66938126	0.081978508	2.542800401	0.000935214	Metallothionein-2
Tac1	0.793824733	1.733664498	0.018111994	4.10606042	9.1731E-05	-0.49399612	-1.40834044	0.212289847	4.106060424	0.006841508	Protachykinin-1
Lingo3	0.510300986	1.424347323	0.000616213	1.05480901	0.00012724	-0.33023196	-1.2572155	7.08543E-07	1.054809009	0.006349737	nogo receptor-interacting protein 3
Lgi2	0.403875622	1.323057371	0.235098845	3.38224578	0.00013609	-0.34006248	-1.26581141	0.327730726	3.382245777	0.000408164	Leucine-rich repeat LGI family member 2
Lrrc10b	1.617177285	3.067742276	0.023598436	1.85710938	0.00014071	-1.44180859	-2.71661211	0.023884469	1.857109381	0.000128865	Leucine-rich repeat-containing 10B
Adgra1	0.431467665	1.348604826	0.027992342	6.42744912	0.00019487	-0.3060218	-1.23629394	0.15376258	6.427449118	0.003604943	Adhesion G protein-coupled receptor A1
Kcnip3	0.378913199	1.300361906	0.359224723	5.61065813	0.00019816	-0.30424257	-1.2347702	0.427514207	5.610658128	0.001009504	Calsenilin
Gpr88	1.181789438	2.268579846	0.028399023	4.29369802	0.0002028	-0.99671839	-1.9954559	0.053506837	4.293698017	0.000434976	Probable G-protein coupled receptor 88
Endod1	0.401659238	1.321026345	0.036044158	6.91829031	0.00029877	-0.30986063	-1.23958795	0.120815147	6.918290312	0.002121449	Endonuclease domain-containing 1 protein
Drd1	0.750260272	1.682096264	0.036609622	4.5094095	0.00030912	-0.4816838	-1.39637245	0.257920656	4.509409504	0.010159282	D(1A) dopamine receptor
B3galt5	0.742991826	1.673643002	0.041753476	5.5295879	0.00037131	-0.43694861	-1.35373805	0.351448985	5.529587902	0.020139848	Beta-1,3-galactosyltransferase 5
Bspry	1.104984262	2.150965316	0.054088978	5.3879884	0.0005116	-0.88619929	-1.84830045	0.100769271	5.387988399	0.001851161	B box and SPRY domain-containing protein
Tgfbr1	0.499936721	1.414151534	0.048805585	5.59125507	0.0005195	-0.36556241	-1.28838379	0.185569224	5.591255071	0.005150765	TGF-beta receptor type-1
Pde10a	0.570173722	1.48470234	0.049369904	6.52733383	0.00053439	-0.50523651	-1.41935602	0.068872835	6.527333828	0.000681213	cAMP-inhibited cGMP 3',5'-cyclic phosphodiesterase 10A
Bcl11b	0.371154636	1.293387556	0.282790375	5.11076137	0.00061413	-0.30657648	-1.23676935	0.472753501	5.110761371	0.001856939	B-cell lymphoma/leukemia 11B
Itpk1	0.43190932	1.34901774	0.188628312	5.10687418	0.00061758	-0.36534244	-1.28818736	0.021723766	5.106874183	0.001461867	Inositol-tetrakisphosphate 1-kinase
Fmo2	1.558165104	2.944790711	0.054088978	1.63880012	0.00061789	-1.07559652	-2.10759333	0.219484807	1.638800121	0.00725537	Dimethylaniline monooxygenase [N-oxide-forming] 2
Alad	0.584341737	1.499354718	0.059349504	5.18436378	0.00071971	-0.37903524	-1.30047192	0.312779425	5.18436378	0.015298306	Delta-aminolevulinic acid dehydratase
Rem2	1.016218106	2.022609915	0.38699176	5.14087121	0.00076213	-0.92450455	-1.8980323	0.411574949	5.140871209	0.000723829	GTP-binding protein REM 2
Serpina9	2.202416099	4.602494825	0.277180923	4.50251459	0.00080926	-1.7577323	-3.38166159	0.137558436	4.502514587	0.002121218	Serpin A9
Uhrf1	1.930561105	3.812034312	0.155245002	-0.5991399	0.00093524	-1.49939721	-2.82724559	0.028311501	-0.5991399	0.0032095	E3 ubiquitin-protein ligase UHRF1
Gng3	0.359651417	1.283115835	0.076898422	5.36825195	0.00095665	-0.41963989	-1.33759364	0.384914487	5.368251951	2.3338E-05	Guanine nucleotide-binding G(I)/G(S)/G(O) gamma-3
Gstm1	0.403841282	1.323025879	0.140500746	3.83323946	0.00115479	-0.30514297	-1.23554107	0.065962145	3.833239462	0.006904183	Glutathione S-transferase Mu 1
Cabp7	0.609924787	1.526179642	0.080904195	4.46943229	0.00124701	-0.40837714	-1.32719204	0.325439241	4.469432294	0.016927542	Calcium-binding protein 7
Spred2	0.414279021	1.332632535	0.023341994	6.51966556	0.00124999	-0.33268176	-1.25935215	0.05139399	6.519665558	0.0043479	Sprouty-related, EVH1 domain-containing protein 2
Rhbdl3	0.563942348	1.478303355	0.082686896	4.62373522	0.00130282	-0.33269512	-1.25936381	0.449638261	4.623735218	0.03620936	Rhomboid-related protein 3
Irf4	1.677638696	3.199039256	0.432170349	0.44410053	0.00136019	-1.19261327	-2.28566389	0.088975051	0.444100535	0.009275355	Interferon regulatory factor 4
Pdk4	0.712518517	1.638662239	0.084760466	3.66977952	0.00138952	-0.45686389	-1.37255493	0.370872154	3.669779518	0.023236769	kinase isozyme 4, mitochondrial
Nr1d2	0.406733222	1.325680598	0.241353165	-0.3500127	0.00148722	-0.31404549	-1.24318887	0.005696831	-0.35001275	0.006816904	Nuclear receptor subfamily 1 group D member 2
Cpm	1.074789921	2.106415322	0.087669521	3.35717755	0.00150021	-0.8758416	-1.83507827	0.158469855	3.357177545	0.003789432	Carboxypeptidase M
Nde1	0.583983636	1.4989826	0.088920822	3.98210605	0.00152561	-0.49712802	-1.41140109	0.140218504	3.982106054	0.002972519	Nuclear distribution protein nudE homolog 1
Map3k13	0.493273927	1.40763562	0.101897888	4.98634092	0.00194644	-0.50424369	-1.4183796	0.05821738	4.98634092	0.000491573	Mitogen-activated protein kinase kinase kinase 13
Chga	0.368441896	1.290957849	0.264897585	4.39865107	0.00220507	-0.3240521	-1.25184166	0.336468552	4.398651073	0.003015882	Chromogranin-A
Eif3j1	1.060685742	2.085922767	0.121940532	3.11351385	0.00264529	-0.66351394	-1.58393588	0.447711277	3.113513849	0.035511256	Eukaryotic translation initiation factor 3 subunit J-A
Strip2	0.466937704	1.382172523	0.123013877	5.41747801	0.00269758	-0.44140054	-1.35792193	0.109954452	5.417478015	0.001780185	Striatin-interacting proteins 2
Il6ra	0.845869036	1.797347087	0.260503716	3.27780322	0.00279749	-0.61505494	-1.53161632	0.150205344	3.277803216	0.015932656	Interleukin-6 receptor subunit alpha
Thrsp	0.794131263	1.73403289	0.129558851	3.78422231	0.00301336	-0.72375543	-1.65147534	0.136351773	3.784222307	0.002699562	Thyroid hormone-inducible hepatic protein
Hhatl	0.617617924	1.534339695	0.054088978	6.57541255	0.00308663	-0.65191704	-1.57125468	0.111582361	6.575412551	0.00061124	Protein-cysteine N-palmitoyltransferase HHAT-like protein
Ndrg1	0.669569158	1.590597885	0.140047157	5.45052526	0.00345322	-0.62888566	-1.54637011	0.126158227	5.450525259	0.002305878	Protein NDRG1
Fa2h	0.967539817	1.955503092	0.140313949	2.57954772	0.00346777	-1.06180627	-2.08754351	0.048360119	2.579547717	0.000376518	Fatty acid 2-hydroxylase
Dusp19	0.495448852	1.409759292	0.02458988	6.82691602	0.00347544	-0.43357261	-1.35057392	0.346839966	6.826916017	0.005048077	Dual specificity protein phosphatase 19
Ciart	0.617018214	1.533702022	0.022484573	4.82647083	0.00349595	-0.65477116	-1.57436621	0.20482125	4.826470825	0.000633995	Circadian-associated transcriptional repressor
Anln	1.132801317	2.192841165	0.140667213	4.99585901	0.00355064	-0.85719358	-1.81151101	0.290212274	4.995859014	0.013257894	Anillin
Kcnab1	0.333965333	1.260473103	0.377246791	3.44234922	0.00392944	-0.26371802	-1.20056875	0.167767725	3.442349219	0.012273145	Voltage-gated potassium channel subunit beta-1
Bcas1	0.931763717	1.90760665	0.149953305	6.06500679	0.0040477	-1.03448994	-2.04838931	0.046874988	6.065006791	0.000357905	Breast carcinoma-amplified sequence 1 homolog
Arrdc3	0.542721791	1.456718173	0.152759314	6.43347189	0.00418916	-0.41516687	-1.3334529	0.31352044	6.433471886	0.015390875	Arrestin domain-containing protein 3
Dlk1	2.206868372	4.616720432	0.245365602	-0.4503917	0.00427701	-1.37522558	-2.59408469	0.357952709	-0.45039172	0.035820789	Protein delta homolog 1
Glp1r	1.746008975	3.354293604	0.264897585	0.78226804	0.00432301	-2.14770623	-4.43122698	0.427514207	0.782268043	0.000174205	Glucagon-like peptide 1 receptor

Carhsp1	0.744445083	1.675329749	0.159547164	4.73882474	0.0045933	-0.61595531	-1.53257249	0.243013404	4.738824744	0.009201002	Calcium-regulated heat stable protein 1
Adora2a	1.557922219	2.944294982	0.159764794	1.08009424	0.0046303	-2.06359478	-4.18026611	0.011798892	1.080094244	3.65652E-05	Adenosine receptor A2a
Taf13	0.450610579	1.366618517	0.28365619	4.63198399	0.0047951	-0.28987031	-1.22253037	0.343301039	4.63198399	0.046517161	Transcription initiation factor TFIID subunit 13
Mki67	0.822192294	1.768090712	0.268925315	1.95949701	0.00479696	-0.81022215	-1.75348143	0.467342756	1.959497012	0.002320316	Proliferation marker protein Ki-67
Npy	0.345131247	1.270266538	0.130477741	3.31985867	0.00480072	-0.47134536	-1.38640173	0.064333584	3.319858666	2.34805E-05	Pro-neuropeptide Y
Rsad1	0.476367473	1.391236292	0.363270338	6.86979245	0.00481033	-0.32057836	-1.24883109	0.140218504	6.869792451	0.037020926	Radical S-adenosyl methionine domain-containing protein 1
Gpx3	1.004856972	2.00674454	0.18328842	2.00455371	0.0050191	-1.10596815	-2.15243273	0.178178738	2.004553713	0.000668328	Glutathione peroxidase 3
Gnpda1	0.467807637	1.383006213	0.351411057	3.67805478	0.00566353	-0.64393277	-1.56258294	0.426861495	3.678054777	3.37157E-05	Glucosamine-6-phosphate isomerase 1
Mobp	1.06904312	2.098041362	0.178319478	8.7455829	0.00576094	-1.05721084	-2.08090462	0.121978214	8.745582897	0.002173063	Myelin-associated oligodendrocyte basic protein
Mei1	0.830349159	1.778115647	0.141609562	5.32237522	0.00608355	-0.77535269	-1.71160843	0.497050097	5.322375218	0.004786071	Meiosis inhibitor protein 1
Ppp1r13b	0.344981252	1.270134477	0.082686896	5.84767843	0.00629647	-0.47848755	-1.39328225	0.403395359	5.847678427	3.15321E-05	Apoptosis-stimulating of p53 protein 1
Cat	0.39829681	1.31795107	0.077211723	6.10583673	0.00643022	-0.51622301	-1.43020605	0.212847918	6.105836726	0.000107013	Crat
Penk	0.79111541	1.730411802	0.189454575	4.73977191	0.00648392	-1.28591986	-2.43837473	0.000622978	4.739771907	8.95365E-07	Proenkephalin-A
Ky	0.98587065	1.980508167	0.401004238	4.90983662	0.0065416	-0.83066514	-1.77850514	0.505369666	4.90983662	0.011394733	Kyphoscoliosis peptidase
Otud7b	0.418782454	1.336798902	0.195298931	6.18713723	0.00693831	-0.35161842	-1.27599123	0.285637608	6.187137229	0.012458444	OTU domain-containing protein 7B
Top2a	1.426199247	2.687377973	0.061704997	1.70325754	0.00744476	-2.00669547	-4.01860692	0.070684463	1.703257537	4.57737E-05	DNA topoisomerase 2-alpha
Dnajb9	0.379521758	1.300910542	0.281743516	6.61477485	0.007509	-0.27957949	-1.21384103	0.132354034	6.614774849	0.030709908	DnaJ homolog subfamily B member 9
Lypd1	0.507352248	1.421439059	0.208742482	4.74644928	0.00816594	-0.67208518	-1.59337426	0.021903733	4.746449276	0.00011215	Ly6/PLAUR domain-containing protein 1
Hnrnpa3	0.363833291	1.286840539	0.384714832	2.76450586	0.00838374	-0.59624222	-1.51177372	0.248584887	2.764505863	2.12311E-06	Heterogeneous nuclear ribonucleoprotein A3
Olfml3	0.663285763	1.583685391	0.21128498	3.48591852	0.00843056	-0.66546581	-1.58608029	0.151896692	3.485918519	0.00350662	Olfactomedin-like protein 3
Ppp1r1b	0.617809101	1.53454303	0.215490373	4.94556648	0.00883641	-0.64207604	-1.56057321	0.134353444	4.94556648	0.002583234	Protein phosphatase 1 regulatory subunit 1B
Drd2	1.322372813	2.500770757	0.218708009	0.86540989	0.00912552	-1.4708387	-2.77182985	0.095663011	0.865409893	0.001328889	D(2) dopamine receptor
Plekhg1	0.51241696	1.42643792	0.218715216	3.97419083	0.00915082	-0.4538383	-1.36967946	0.270531959	3.974190833	0.011117752	Pleckstrin homology domain-containing, family G
Spock3	0.450480047	1.366494873	0.219309006	6.26460604	0.00919985	-0.50769051	-1.42177238	0.090627086	6.264606036	0.001184482	Testican-3
Wipf3	0.427674485	1.345063688	0.221035622	5.3451641	0.0093008	-0.6430786	-1.56165807	0.006916144	5.3451641	1.67739E-05	WAS/WASL-interacting protein family member 3
Vcan	0.362787869	1.285908392	0.301902499	5.64422057	0.00939248	-0.33953467	-1.2653484	0.088975051	5.644220569	0.007740279	Versican core protein
Cldn11	0.812016044	1.75566312	0.223577029	5.96413009	0.00958976	-0.73482121	-1.66419122	0.240958586	5.96413009	0.008982512	Claudin-11
Zcchc24	0.349987461	1.274549549	0.223771607	6.04367643	0.00961611	-0.33230361	-1.2590221	0.212289847	6.04367643	0.006863539	Zinc finger CCHC domain-containing protein 24
Aldh1a1	0.434085407	1.351054064	0.225167683	5.23297139	0.00977935	-0.89619134	-1.86114614	6.12593E-06	5.232971387	4.67733E-09	Retinal dehydrogenase 1
Tppp3	0.483943704	1.398561507	0.231449933	4.4773108	0.01020812	-0.36404152	-1.28702629	0.435338022	4.477310797	0.033328671	Tubulin polymerization-promoting protein family member 3
Mal	0.920057247	1.892190375	0.233602207	6.97251653	0.01041846	-0.63371466	-1.55155481	0.503428554	6.972516525	0.048867721	Myelin and lymphocyte protein
Ncapg2	0.55539874	1.469574746	0.239830899	3.38130469	0.01055612	-0.47022485	-1.38532536	0.131393922	3.381304693	0.017233236	Condensin-2 complex subunit G2
Gpr83	0.412218115	1.330730214	0.235098845	4.66558129	0.01057293	-0.35213618	-1.27644925	0.323364703	4.665581288	0.016469597	Probable G-protein coupled receptor 83
Ugt8a	0.740415496	1.670656919	0.237231965	5.8995163	0.0107916	-0.84643567	-1.79805315	0.090359107	5.899516304	0.001172862	2-hydroxyacylsphingosine 1-beta-galactosyltransferase
Peg10	0.573926281	1.48856919	0.048471331	2.13767769	0.0111882	-0.62382262	-1.54095274	0.111582361	2.137677695	0.002472672	Retrotransposon-derived protein PEG10
Rgs9	0.953274988	1.936263085	0.240106056	2.93402948	0.01125851	-0.91279628	-1.88269106	0.212289847	2.934029477	0.006873802	Regulator of G-protein signaling 9
Selplg	0.456705804	1.37240454	0.434597552	7.57841134	0.01145094	-0.39207948	-1.31228355	0.140599742	7.578411337	0.017241183	P-selectin glycoprotein ligand 1
Gins2	1.598889119	3.029099816	0.325551185	-0.161253	0.01165941	-1.29623346	-2.45586876	0.433939744	-0.16125297	0.021414463	DNA replication complex GINS protein PSF2
Mag	0.817349387	1.762165451	0.245490554	6.24588779	0.0116874	-0.86708605	-1.82397512	0.138488759	6.245887795	0.002838533	Myelin-associated glycoprotein
Ckap2	1.476958126	2.783611989	0.190142047	1.00113899	0.0118242	-1.946826	-3.85525424	0.273978107	1.001138989	0.000360343	Cytoskeleton-associated protein 2
Tspan2	0.643002024	1.561575175	0.257844695	6.08373628	0.01299357	-0.6055593	-1.52156852	0.245790015	6.083736285	0.009402099	Tetraspanin-2
Gng7	0.393280928	1.313376847	0.14829128	6.76915496	0.01305676	-0.55909163	-1.47334126	0.284351266	6.769154961	0.000106285	Guanine nucleotide-binding G(I)/G(S)/G(O) gamma-7
Mylk	0.526892723	1.440822607	0.391590661	7.11824527	0.01329136	-0.56903198	-1.48352782	0.301527182	7.11824527	0.003415141	Myosin light chain kinase, smooth muscle
Foxo6	0.586354374	1.501447858	0.295911299	3.2965222	0.01346889	-0.50261924	-1.41678342	0.431238071	3.296522197	0.020087265	Forkhead box protein O6
Hist1h2bm	0.963918832	1.950601177	0.01967957	-1.8561166	0.01395122	-0.75744194	-1.69049053	0.004154879	-1.85611662	0.03195591	Histone H2B type 1-M
Tmem119	0.420060232	1.337983414	0.258407875	5.08861396	0.01399307	-0.36803063	-1.29058989	0.021723766	5.08861396	0.01812549	Transmembrane protein 119
Slc7a1	0.275755962	1.210628284	0.246883791	-0.2733985	0.01423017	-0.27076345	-1.20644609	0.046918378	-0.27339852	0.008440146	High affinity cationic amino acid transporter 1
Pnoc	0.732528131	1.661548183	0.431248816	6.49545084	0.01461485	-0.55651493	-1.47071217	0.448890923	6.495450844	0.040783605	Prepronociceptin
Tmx2	0.298623016	1.229969905	0.161972847	5.95733842	0.01473915	-0.2821753	-1.21602703	0.008860909	5.957338418	0.011321423	Thioredoxin-related transmembrane protein 2
Scn4b	0.511932939	1.425959432	0.270878548	6.50587027	0.01483903	-0.45083239	-1.36682865	0.331036293	6.505870275	0.017633463	Sodium channel subunit beta-4
Esrrg	0.376546727	1.298230654	0.422044386	3.96654444	0.01551167	-0.42573079	-1.34325275	0.000452131	3.966544438	0.00275914	Estrogen-related receptor gamma
Cenpf	1.015625711	2.021779568	0.432078334	2.76678829	0.01595742	-1.29648955	-2.45630472	0.456677796	2.766788291	0.000857403	Centromere protein F
Ina	0.327824153	1.255118998	0.270096523	6.53626939	0.01607071	-0.37365391	-1.29563011	0.272804642	6.536269387	0.002518815	Alpha-internexin
Cln8	0.342949951	1.268347398	0.140313949	3.89271455	0.01619392	-0.26477258	-1.20144665	0.18357956	3.892714547	0.041701679	Protein CLN8
Rassf5	0.364656713	1.287575217	0.177853927	3.85235625	0.01630721	-0.32532233	-1.25294434	0.011085362	3.852356254	0.01888811	Ras association domain-containing protein 5
Zfp462	0.301088191	1.232073387	0.415730143	6.32237558	0.01664349	-0.36378388	-1.28679647	0.010768726	6.322375582	0.001524877	Zinc finger protein 462
Mbp	0.793492132	1.733264863	0.287909056	9.92538278	0.01684911	-0.77627325	-1.71270092	0.241769006	9.925382784	0.009095539	Myelin basic protein
Stmn4	0.266318083	1.202734396	0.379833435	5.15728598	0.01704823	-0.30901482	-1.23886142	0.102883002	5.157285985	0.002360827	Stathmin-4
Cd9	0.513738021	1.427744692	0.290451735	3.94090639	0.01728276	-0.53702851	-1.45098088	0.202309571	3.940906388	0.006199206	CD9 antigen
Abcc9	0.469241804	1.384381727	0.383690305	2.94964226	0.01785513	-0.3862259	-1.30696989	0.097479869	2.949642264	0.032644008	ATP-binding cassette sub-family C member 9
Tmcc3	0.507373648	1.421460144	0.297199984	4.96867055	0.01822172	-0.53515763	-1.44910047	0.197377467	4.968670555	0.0059628	Transmembrane and coiled-coil domains protein 3
Rpl15	0.299334706	1.230576806	0.163856721	1.64244742	0.01841017	-0.44180816	-1.35830566	0.067946668	1.642447422	0.000132088	60S ribosomal protein L15
Csrp1	0.40629776	1.325280515	0.299196376	7.39160977	0.01866591	-0.35036899	-1.27488666	0.38447008	7.39160977	0.025156127	Cysteine and glycine-rich protein 1
Pkp4	0.292680299	1.224913861	0.242814522	4.01993878	0.01875633	-0.31469061	-1.2437449	0.327730726	4.019938777	0.005362167	Plakophilin-4

Tmsb10	0.385512667	1.306323912	0.064174814	-0.2775448	0.01905123	-0.43371815	-1.35071018	0.120815147	-0.27754476	0.003933037	Thymosin beta-10
Ksr1	0.306143942	1.236398614	0.028101583	6.89375219	0.01912954	-0.38788573	-1.30847443	0.084559815	6.893752194	0.001128712	Kinase suppressor of Ras 1
Wisp1	0.921353394	1.893891122	0.304387263	2.55811671	0.01935829	-1.48285915	-2.79502105	0.011085362	2.558116713	3.3856E-05	WNT1-inducible-signaling pathway protein 1
Gstm7	0.547278559	1.461326507	0.43003852	3.88421184	0.01993778	-0.42706337	-1.34449405	0.491901446	3.884211842	0.045794246	Glutathione S-transferase Mu 7
Lpin3	1.16455703	2.241643769	0.440847945	0.85207614	0.02135647	-0.90348152	-1.87057462	0.458606011	0.852076141	0.046887285	Phosphatidate phosphatase LPIN3
Il1rl2	1.314012535	2.48632094	0.387320673	0.79738601	0.02231265	-1.09861274	-2.14148673	0.064333584	0.797386008	0.033150541	Interleukin-1 receptor-like 2
Havcr2	0.696844983	1.62095606	0.336699542	2.98295812	0.02268621	-0.78211256	-1.71964713	0.334032827	2.982958118	0.005243007	Hepatitis A virus cellular receptor 2 homolog
Pacs2	0.341591664	1.26715382	0.331739847	6.11497814	0.02329006	-0.46422303	-1.37957418	0.068872835	6.114978137	0.000684303	Phosphofurin acidic cluster sorting protein 2
Sema4d	0.28813929	1.221064399	0.431248816	4.38256357	0.02383087	-0.47153341	-1.38658245	0.451978101	4.382563568	5.2237E-05	Semaphorin-4D
Trabd2b	0.481733947	1.396420989	0.26181108	2.62945132	0.02430088	-0.46316053	-1.37855854	0.351448985	2.629451319	0.017871162	Metalloprotease TIKI2
Srm	0.29231234	1.224601487	0.327992568	1.92197108	0.02512445	-0.44232689	-1.35879413	0.186828065	1.921971078	0.000219035	Tyrosine-protein kinase Srms
Myrf	0.546167014	1.460201042	0.342607268	3.34104917	0.02528967	-0.79193455	-1.73139458	0.048360119	3.341049168	0.00037822	Myelin regulatory factor
Trf	0.797701944	1.73832995	0.348862986	3.50460573	0.0265584	-0.7409604	-1.67128805	0.364674852	3.504605733	0.022054069	Interleukin-5
Pip5k1b	0.464490869	1.379830322	0.178278352	9.31573618	0.02704234	-0.41137241	-1.32995037	0.316078576	9.315736178	0.03151854	Phosphatidylinositol 4-phosphate 5-kinase type-1 beta
Tns3	0.301208579	1.232176204	0.201730278	4.98236864	0.02812166	-0.26810607	-1.20422592	0.420772985	4.982368637	0.032027563	Tensin-3
Tenm2	0.280161361	1.214330696	0.297721486	6.12343132	0.02884558	-0.34612188	-1.27113907	0.023940743	6.123431324	0.002960652	Teneurin-2
Tmem125	0.782632406	1.720266887	0.363399869	2.07122469	0.02895499	-1.05632674	-2.07962981	0.091844017	2.071224691	0.001221012	Transmembrane protein 125
Il33	0.701581612	1.626286698	0.363645132	4.76519551	0.02928434	-0.90750036	-1.87579265	0.107889784	4.765195514	0.001729919	Interleukin-33
Pde8a	0.499079026	1.413311058	0.371431468	3.56233891	0.0307534	-0.60453673	-1.52049042	0.164459878	3.562338911	0.004092107	High affinity 3',5'-cyclic phosphodiesterase 8A
Plin3	0.494177929	1.40851793	0.080904195	5.89354665	0.031797	-0.60036875	-1.51610403	0.170433106	5.893546647	0.004212089	Perilipin-3
Tsc22d4	0.291331661	1.223769341	0.286094402	5.56791057	0.03237924	-0.39454339	-1.31452665	0.102883002	5.567910567	0.001524626	TSC22 domain family protein 4
Ly86	0.502366057	1.416534812	0.161972847	1.76221783	0.03301822	-0.69267286	-1.6162752	0.126622147	1.76221783	0.001365988	Lymphocyte antigen 86
Rassf2	0.346714944	1.27166172	0.384108381	5.88783521	0.03313648	-0.42272295	-1.34045515	0.168121178	5.887835214	0.004243616	Ras association domain-containing protein 2
Dach1	0.617215072	1.533911313	0.109588838	7.09042997	0.03324461	-0.68352582	-1.60606003	0.140323755	7.090429968	0.009612917	Dachshund homolog 1
Slc5a7	0.846888737	1.798617907	0.280419897	0.74735332	0.0336507	-1.13698979	-2.19921674	0.078884622	0.747353319	0.001994563	High affinity choline transporter 1
Cntn2	0.264180181	1.200953407	0.185683358	5.85404416	0.03376442	-0.37060764	-1.29289726	0.010768726	5.854044158	0.00105513	Contactin-2
Car14	0.776310678	1.712745362	0.38699176	2.09850582	0.0339062	-0.8704978	-1.82829365	0.239219636	2.098505815	0.008842477	Caspase recruitment domain-containing protein 14
Kdm5c	0.316150804	1.245004368	0.38699176	6.30547796	0.03408383	-0.36996823	-1.29232437	0.20482125	6.305477955	0.006330217	Lysine-specific demethylase 5C
C1qa	0.371556819	1.293748168	0.07006504	6.10804728	0.03410461	-0.34808071	-1.27286614	0.008860909	6.108047277	0.029095844	Complement C1q subcomponent subunit A
Plpp2	0.83640473	1.785594798	0.386351381	0.90233914	0.0342331	-1.26200464	-2.39828754	0.117483967	0.902339136	0.000610763	Phospholipid phosphatase 2
Cryab	0.505875502	1.419984815	0.387320673	5.06425347	0.03430707	-0.47063711	-1.38572128	0.415921989	5.064253467	0.029594546	Alpha-crystallin B chain
Ermn	0.748139439	1.679625317	0.391590661	5.7042911	0.03521586	-0.74273225	-1.67334191	0.350816519	5.704291097	0.019913907	Ermin
Pde1b	0.270007867	1.205814403	0.299361829	7.96228994	0.03535132	-0.28608386	-1.21932597	0.187423319	7.962289943	0.014169604	Ca/CaM-dependent 3',5'-cyclic nucleotide PDE 1B
Cplx3	0.595272729	1.510758155	0.401004238	3.28583456	0.03710167	-0.7799138	-1.71702828	0.136302273	3.285834555	0.002650747	Complexin-3
Kntc1	1.347997898	2.545586161	0.320851391	-0.0738416	0.03936841	-1.20123293	-2.29936091	0.496920153	-0.07384159	0.040722507	Kinetochore-associated protein 1
Kcnj10	0.297589639	1.229089215	0.416029352	3.42364372	0.04063008	-0.55016011	-1.46424819	0.451978101	3.42364372	3.07857E-05	ATP-sensitive inward rectifier potassium channel 10
Hdhd3	0.486953047	1.401481836	0.161972847	3.95743429	0.04074043	-0.45089893	-1.36689169	0.495599099	3.957434287	0.037112174	Haloacid dehalogenase-like protein 3
Hapln2	0.679049842	1.601084934	0.416210389	3.10982804	0.04092003	-0.73458441	-1.66391808	0.305012068	3.109828042	0.014452627	Hyaluronan and proteoglycan link protein 2
Pappa2	0.373729164	1.295697703	0.289174273	6.71933556	0.04210018	-0.84620671	-1.79776782	0.127486868	6.719335565	5.88897E-07	Pappalysin 2
Vsnl1	0.326257454	1.253756738	0.426036524	8.38615942	0.04299592	-0.42572384	-1.34324628	0.152157996	8.386159422	0.00353376	Visinin-like protein 1
Synpo	0.320841389	1.249058796	0.429624692	7.34935941	0.04397986	-0.32586159	-1.25341277	0.377398091	7.349359413	0.024120255	Synaptopodin
Pcyox1l	0.379750117	1.301116475	0.34991453	5.67815657	0.04434621	-0.34507828	-1.2702199	0.467450926	5.678156568	0.04533491	Prenylcysteine oxidase-like
Slc25a33	0.287529438	1.220548343	0.010414325	-0.0506537	0.04522019	-0.27437077	-1.20946646	0.140323755	-0.05065368	0.037034961	Solute carrier family 25 member 33
Arsj	0.459985655	1.375528141	0.266471617	5.63899987	0.04588998	-0.43622492	-1.35305916	0.234314025	5.638999872	0.038478668	Arylsulfatase J
Il3ra	0.906300911	1.874233773	0.154433309	-0.6068847	0.04603426	-1.37968762	-2.60212022	0.448890923	-0.60688469	0.001131622	Interleukin-3 receptor subunit alpha
Ido1	2.407626783	5.30600775	0.084358057	-0.4707316	0.04694285	-2.16131275	-4.47321701	0.24439736	-0.47073163	0.04285676	Indoleamine 2,3-dioxygenase 1
Mog	0.788271903	1.727004573	0.434597552	4.76059643	0.04694317	-0.99334204	-1.99079138	0.186876178	4.760596429	0.00526801	Myelin-oligodendrocyte glycoprotein
Selenow	0.291514973	1.223924846	0.221735236	4.88569975	0.04704879	-0.39441199	-1.31440693	0.226164439	4.885699747	0.003043749	Selenoprotein W
Sox10	0.609978115	1.526236056	0.435963351	2.80492153	0.0476586	-1.15679751	-2.22961947	0.012935539	2.804921529	4.12496E-05	Transcription factor SOX-10
Krt80	0.652827442	1.572246519	0.440847945	2.42784974	0.04950898	-1.06110215	-2.08652492	0.057639482	2.427849738	0.000481516	Keratin, type II cytoskeletal 80
Klk8	0.790582551	1.729772793	0.069503499	-0.4363061	0.04969343	-0.75911802	-1.69245564	0.144362647	-0.43630613	0.038826514	Kallikrein-related peptidase 8

Suppl. Table 3 Classification of genes down-regulated in P301S Tau transgenic mice and reversed by UNC0642 treatment

17	14	13	13	10	10	7	19
**cytoskeleton/cell adhesion/extracellular matrix protein**	**kinase/enzyme**	**cell proliferation /differentiation/ division/development**	**nucleotide-binding/transcription regulator**	**receptor/signaling molecule**	**ion channel /transporter/synaptic transmission**	**defense/immunity protein**	**others**
Cldn5	Hhipl1	Frzb	Zfp386	Leng8	Col23a1	Hmcn1	Snhg11
Fn1	Pla2g4e	Btg2	Snrnp70	Akap13	Slc16a1	Otud7a	Spaca6
Ccdc85c	Malt1	Evc2	Rnpc3	Rab40b	Trpm4	Tbkbp1	BC005561
Col5a1	Smpd4	Cspp1	Luc7l3	Wsb1	Bloc1s6	Cd93	Wdr90
Postn	Hars2	Amn1	Zbtb49	Grin2c	Slc9a2	Rnasel	Clba1
Fmod	Sphk1	Spag5	Wtip	Tnip2	Clcn1	Gbp11	Vstm4
Hspg2	Alg6	Notch3	Ing4	Miip	Fxyd5	Il13ra2	Ccdc73
Col11a1	Nsun5	Haus4	Abra	Reck	Crabp2		Lrrc29
Flnb	Etnppl	Bmp4	Mamstr	Ptgfr	Slc12a7		Pdss1
Col4a2	Setd4	Adam8	Nxf7	Gpr171	Stx19		Tspan18
Olfml2a	L3hypdh	Ppp1r42	Perm1				Grrp1
Mmp14	Chst14	Mab21l1	Zscan30				Fam228a
Kif26a	Chst9	Prrx2	Zfp984				Fam181a
Col7a1	C1rl						
Cdh1							
Adgrg3							
Actl10							

Suppl. Table 4 Classification of genes up-regulated in P301S Tau transgenic mice and reversed by UNC0642 treatment								
40	31	23	20	16	11	8	7	17
**receptor/signaling molecule**	**kinase/enzyme**	**nucleotide-binding/transcription regulator**	**cytoskeleton/cell adhesion/extra-cellular matrix**	**apoptosis/oxidative stress/immune response**	**ion channel /transporter/synaptic transmission**	**cell proliferation/ differentiation/ division/development**	**myelination**	**others**
Gpr88	Ugt8a	Rpl3	Anln	Il33	Scn4b	Spock3	Mobp	Ighm
Tspan2	B3galt5	Carhsp1	Ermn	Ndrg1	Cplx3	Csrp1	Mal	Plin4
Penk	Cpm	Thrsp	Cldn11	Cryab	Bspry	Krt80	Mbp	Per2
Ppp1r1b	Pde10a	Eif3j1	Haus2	Pacs2	Plin3	Ncapg2	Bcas1	Trf
Arrdc3	Otud7b	Sox10	Hapln2	Rassf2	Kcnab1	Mei1	Mag	Per1
Rgs9	Pdk4	Kdm5c	Mt1	Spred2	Kcnip3	Mki67	Mog	Lrrc10b
Drd1	Fa2h	Endod1	Strip2	Chga	Kcnj10	Cenpf	Myrf	Rhbdl3
Tmcc3	Alad	Ciart	Tppp3	Dnajb9	Abcc9	Kntc1		Plekhg1
Tac1	Fmo2	Peg10	Nde1	Selplg	Slc25a33			Zcchc24
Wisp1	Pde8a	Dach1	Synpo	Il6ra	Slc7a1			Lingo3
Sst	Aldh1a1	Hnrnpa3	Wipf3	Ppp1r13b	Slc5a7			Lgi2
Adora2a	Map3k13	Bcl11b	Adgra1	C1qa				Hhatl
Lypd1	Car14	Selenow	Olfml3	Ly86				Pkp4
Cabp7	Gstm1	Top2a	Cthrc1	Havcr2				Rsad1
Tgfbr1	Cat	Rpl15	Ina	Il1rl2				Pcyox1l
Cd9	Itpk1	Taf13	Vcan	Il3ra				Hdhd3
Vsnl1	Dusp19	Zfp462	Cntn2					Serpina9
Tmem125	Tmx2	Tsc22d4	Stmn4					
Mt2	Mylk	Foxo6	Tmsb10					
Drd2	Gpx3	Hist1h2bm	Ckap2					
Gpr83	Gstm7	Gins2						
Nr1d2	Pip5k1b	Uhrf1						
Gng7	Gnpda1	Irf4						
Tmem119	Pappa2							
Cln8	Srm							
Npy	Acaa1b							
Tns3	Arsj							
Ksr1	Plpp2							
Pde1b	Klk8							
Gng3	Lpin3							
Rem2	Ido1							
Tenm2								
Rassf5								
Esrrg								
Pnoc								
Trabd2b								
Sema4d								
Ky								
Glp1r								
Dlk1								
